# Secreted Glycoside Hydrolase BcGH61 From *Botrytis cinerea* Induces Cell Death by the Apoplastic Location and Triggers Intracellular Immune Perception

**DOI:** 10.1111/mpp.70199

**Published:** 2025-12-30

**Authors:** Wenjun Zhu, Ziyao Wang, Can Zheng, Min Fang, Binbin Huang, Xiaofei Nie, Yong Liang, Zhaoxia Li, Kai Bi

**Affiliations:** ^1^ School of Life Science and Technology Wuhan Polytechnic University Wuhan City Hubei Province China; ^2^ School of Plant Sciences and Food Security, Faculty of Life Sciences Tel Aviv University Tel Aviv Israel; ^3^ College of Marine and Biological Engineering Yancheng Institute of Technology Yancheng Jiangsu Province China

**Keywords:** BcGH61, *Botrytis cinerea*, CDIPs, defence responses, NbHrBP1, pathogenicity

## Abstract

Cell death‐inducing proteins (CDIPs) are critical mediators of infection in the necrotrophic pathogen *Botrytis cinerea*, enabling host colonisation across its broad plant host range. Here, we identified a novel plant CDIP from *B. cinerea* , BcGH61, that exhibits dual activity: triggering extensive plant cell death while simultaneously promoting host defence responses. Functional analysis demonstrates that its full cell death‐inducing capacity requires its apoplastic compartmentalisation, intact glycoside hydrolase activity, two conserved cysteine residues, and specific structural motifs spanning residues 193–240. Notably, this phytotoxic activity operates through a mechanism independent of the canonical immune coreceptors BAK1 and SOBIR1. During pathogenic invasion, *bcgh61* demonstrates upregulated expression. While the *bcgh61* gene deletion mutant displays no significant developmental defects, it exhibits severely attenuated virulence, establishing BcGH61 as an essential pathogenicity factor. Furthermore, we identified NbHrBP1, a *Nicotiana benthamiana* hypersensitive response‐binding protein, as a direct interactor of BcGH61 during infection, functioning in intracellular immune perception but not in cell death induction. Taken together, our results provide evidence that BcGH61 is a novel critical virulence factor for establishing infection in host plant by its phytotoxic activity and underscore the role of NbHrBP1‐based plant surveillance system in recognising fungal secreted proteins as a pivotal defence strategy against necrotrophic pathogens.

## Graphical abstract

We present a dual‐action virulence paradigm for BcGH61: Extracellularly, BcGH61 glycoside hydrolase degrades cell walls, releasing DAMPs that activate PRR‐mediated cell death. Intracellularly, it targets chloroplasts via NbHrBP1 interaction at plastoglobule‐thylakoid interfaces, paradoxically activating defence signalling while priming immunity‐related transcription to generate necrotic microenvironments, enabling colonisation through spatially partitioned mechanisms.

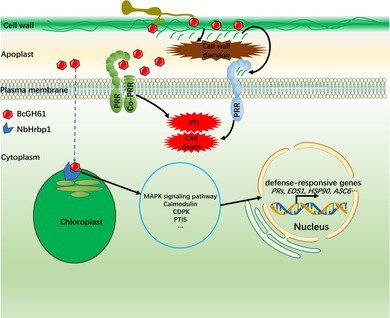

## 1 Introduction

*Botrytis cinerea*, a necrotrophic fungal pathogen with a broad host range, is responsible for devastating grey mould disease in over a thousand plant species, incurring substantial global economic losses (Valero‐Jiménez et al. 2019). Recognised as the second most scientifically and economically significant fungal plant pathogen, *B. cinerea* serves as a model fungus for studying plant–necrotrophic pathogen interactions (Dean et al. 2012). There are three main infection stages that facilitate disease progression: an initial phase marked by localised necrotic tissue formation without direct host penetration, thereby evading early plant immune recognition; an intermediate phase characterised by accelerated host cell death expansion; and a terminal phase defined by aggressive lesion spread (Bi et al. 2021). Critical to this strategy are early‐secreted cell death‐inducing factors, which generate necrotic microenvironments that the pathogen exploits as footholds for subsequent colonisation (Tyler et al. 2011). While plant cell wall‐degrading enzymes (PCWDEs) are known contributors to early infection, recent studies highlight the equally vital role of diverse cell death‐inducing proteins (CDIPs) in this phase (Bi et al. 2023). However, the functional redundancy among CDIPs and their molecular interplay with host targets remain poorly characterised, leaving significant gaps in our understanding of *B. cinerea* pathogenesis (Leisen et al. 2022).

Lacking specialised immune cells and adaptive immunity, but sessile plants have evolved a series of preformed defence barriers and sophisticated innate immune system to counteract invasions by all classes of pathogens (Jones and Dangl 2006; Spoel and Dong 2012; Zipfel 2014; Yu et al. 2017). Over millions of years, the co‐evolutionary ‘arms race’ between host plants and pathogens has led to the development of a two‐tiered innate immune system in plants, consisting of pattern‐triggered immunity (PTI) and effector‐triggered immunity (ETI) (Yuan et al. 2021). On the plants plasma membrane, multiple pattern recognition receptors (PRRs) recognise pathogen‐associated molecular patterns (PAMPs) to initiate PTI. This activation triggers downstream signalling pathways, resulting in the production of reactive oxygen species (ROS), stomatal closure, callose deposition, activation of mitogen‐activated protein kinases (MAPKs), and the synthesis of defence‐related hormones (Ahuja et al. 2012; Albert et al. 2020; Yuan et al. 2021). In addition, many of these PRRs rely on coreceptors such as BRI1‐associated kinase‐1 (BAK1) and suppressor of BIR1‐1 (SOBIR1) to regulate PRR‐mediated immune signalling (van der Burgh et al. 2019).

To successfully infect plants, pathogens produce a diverse arsenal of cocktail effectors that facilitate host colonisation through multiple mechanisms. These effectors enable pathogens to overcome physical barriers, suppress or evade immune recognition, and acquire nutrients from host tissues (Macho and Zipfel 2015). Among these effectors, CDIPs play a critical role in virulence by triggering host cell death. Interestingly, many CDIPs are also recognised as PAMPs, which activate plant defence responses and enhance systemic immunity in uninfected tissues (Bi et al. 2023). In general, CDIPs can be classified into two categories: noncatalytic CDIPs, which lack a catalytic domain, and catalytic CDIPs, which are secreted enzymes that directly induce plant cell death (Bi et al. 2021).

The plant cell wall, primarily composed of carbohydrates such as cellulose and pectic polysaccharides, plays a critical role in maintaining structural integrity and providing a defence barrier against pathogen invasion (Popper et al. 2011; Zhang et al. 2021). Many necrotrophic and hemibiotrophic pathogenic fungi, as well as oomycetes, contain around 300 genes encoding glycoside hydrolases (GH) proteins, which are key components of their virulence arsenal (Zerillo et al. 2013; Zhao et al. 2013). As the largest class of carbohydrate‐active enzymes (CAZymes), secreted GH proteins in plant pathogens perform diverse functions. Recent studies have revealed that some catalytic CDIPs from *B. cinerea* , typically GHs, are involved in breaking down plant cell wall and in some cases their necrosis activity was unrelated to their enzymatic activity (Yang et al. 2018; Jeblick et al. 2023). For instance, BcXYG1, a secreted GH12 protein from *B. cinerea* , triggers cell death through the co‐receptors BAK1 and SOBIR1 and activates defence responses independently of its enzymatic activity (Zhu et al. 2017). Similarly, the *Phytophthora sojae* GH12 protein PsXEG1 and the *Ustilaginoidea virens* GH42 protein UvGHF1 function as key virulence factors while also being recognised as PAMPs that trigger immune responses (Ma et al. 2015; Zou et al. 2023). Additionally, BcCrh1 and BcCrh4, two GH16 transglycosylases from *B. cinerea* , and VdEIX3, a GH11 protein from *Verticillium dahliae*, have been shown to induce plant cell death and activate immune responses (Bi et al. 2021; Yin et al. 2021; Liang et al. 2023).

Based on recent findings that emphasise the plant apoplast as a critical battleground for plant–pathogen interactions (De Wit 2016), we analysed the secretome of bean leaves infected by *B. cinerea* spore suspension to characterise CDIPs and screen potential candidates (Zhu et al. 2017). In this study, we identified BcGH61 as a fungal‐secreted effector that induces plant cell death, critically contributes to pathogen virulence, and activates host defence responses via NbHrBP1‐mediated intracellular immune perception. Crucially, we established that BcGH61‐triggered cell death operates independently of the canonical immune co‐receptors BAK1 and SOBIR1, while simultaneously demonstrating the requirement of its GH activity, conserved cysteine residues, and specific peptides for phytotoxic effects. Furthermore, we identified NbHrBP1, a *Nicotiana* *benthamiana* hypersensitive response (HR)‐binding protein, as a direct interactor of BcGH61 that mediates intracellular immune perception but not cell death induction during infection. Overall, our findings provide an experimental framework for investigating the molecular interactions between *B. cinerea* and its host plants, shedding light on the pathogen's virulence mechanisms and their impact on host immunity.

## 2 Results

### 2.1 The Secretory Protein BcGH61 From *B. cinerea* Localises to the Apoplast in Plant Cells

**FIGURE 1:**
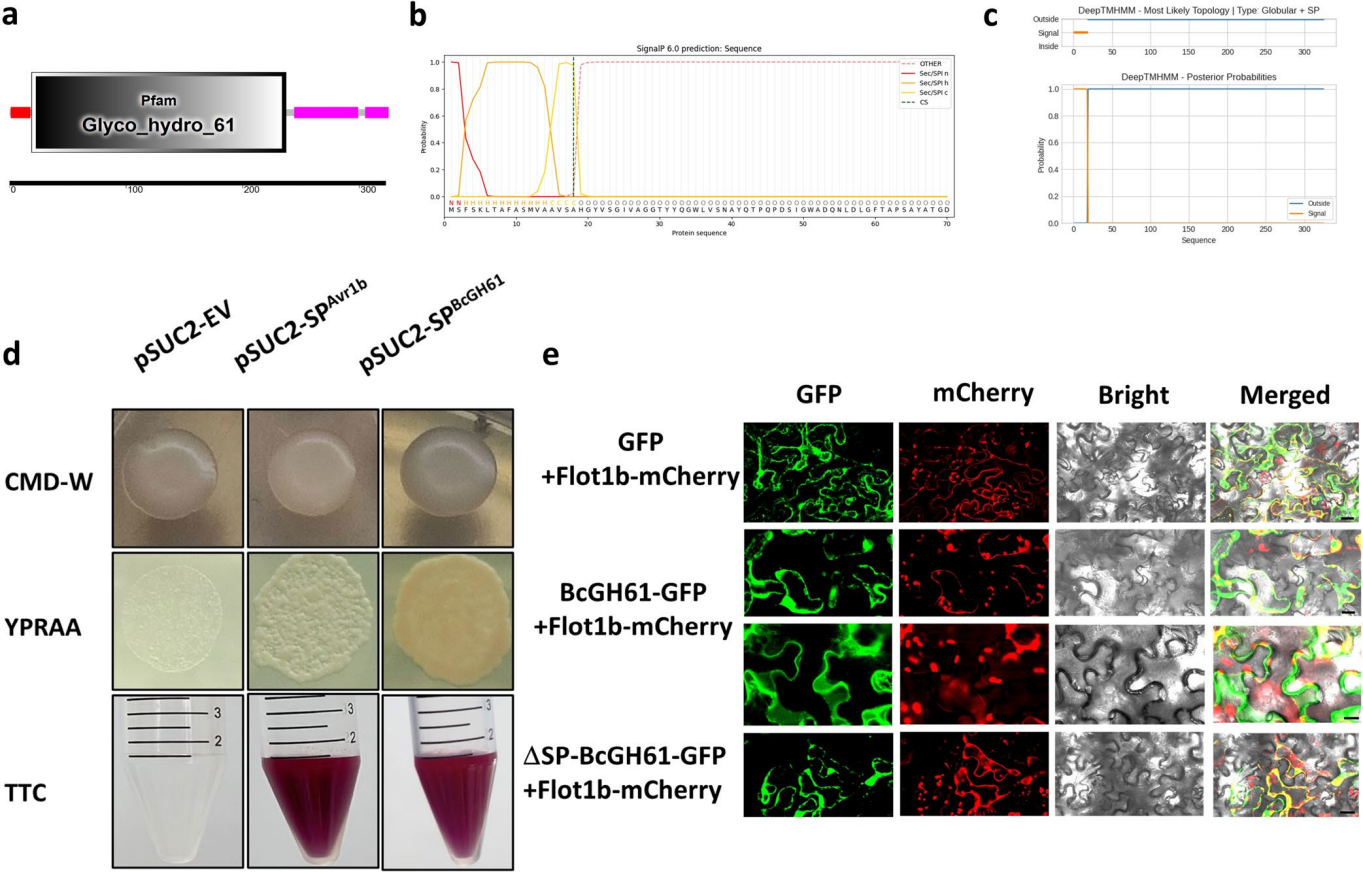
GH61 domain containing protein Bcin09g06750 has a functional signal peptide. (a) Schematic presentation of predicted signal peptides (SP, red), GH61 domain (grey) and function unknown region (pink) of BcGH61 proteins. (b) The SignalP 6.0 server predicts the presence of N‐terminal SP and the location of cleavage site of BcGH61. (c) The transmembrane domain prediction of BcGH61. The transmembrane domain prediction for the BcGH61 using DeepTMHMM. DeepTMHMM predicts transmembrane domain containing proteins using deep neural networks. (d) Functional validation of SP of BcGH61 using the yeast invertase secretion assay. Expression of N‐terminal SP fragment of BcGH61 in mutant yeast strain YTK12 that lacks a functional secreted invertase gene. Yeast YTK12 strains carrying the BcGH61 and positive control (Avr1b) SP fragments fused in frame to the invertase gene in the pSUC2 vector are able to grow in both the CM−W medium (with sucrose, yeast growth even in the absence of invertase secretion) and YPRAA medium (with raffinose instead of sucrose, growth only when invertase is secreted), and reduces 2,3,5‐triphenyltetrazolium chloride (TTC) to red‐coloured triphenylformazan, indicating secretion of invertase. Transformation control: Selection of transformed yeasts with a plasmid containing tryptophan synthesis genes that can grow on CMD−W medium. YTK12 strain carrying the empty pSUC2 vector serves as negative control. (e) Subcellular localisation of GFP‐fusion proteins was analysed in *Nicotiana benthamiana* leaves transiently co‐expressed with the plasma membrane marker Flot1b‐mCherry. Leaf samples were collected 2 days after agroinfiltration and subjected to plasmolysis by incubation in 0.8 M mannitol for 20 min. The fluorescences of eGFP and mCherry were then visualised using a confocal laser scanning microscope. The “Merged” panels show the overlay of fluorescence signals with bright‐field images. Scale bars represent 20 μm in the top and bottom panels, and 10 μm in the middle panels.

The *bcgh61* gene (*Bcin09g06750*), contains an open reading frame of 2198 bp encoding a 325‐amino acid protein featuring a conserved GH 61 domain (GH61) spanning residues 19–237 (*E*‐value 1.3e–69) (Figure 1a). Bioinformatics analysis predicted an N‐terminal signal peptide (SP, residues 1–18) and absence of transmembrane domains (Figure 1b,c), consistent with our previous secretome analysis identifying BcGH61 as a secreted protein in *B. cinerea* (Zhu et al. 2017). To experimentally validate signal peptide functionality, we performed a yeast signal trap assay by cloning the BcGH61 signal sequence into pSUC2 (pSUC2:SP^BcGH61^). Both control (pSUC2:EV) and experimental constructs transformed into yeast YTK12 showed growth on CMD−W plates, confirming successful transformation. However, only transformants carrying either pSUC2:SP^BcGH61^ or the positive control pSUC2:SP^Avr1b^ exhibited robust growth on YPRAA medium containing raffinose as the carbon source, and produced insoluble red triphenylformazan from 2,3,5‐triphenyltetrazolium (TTC, Figure 1d). Because both phenotypes depend on invertase secretion, these results demonstrate that the BcGH61 SP effectively mediates secretory activity.

We further investigated BcGH61 subcellular localisation in planta using *Agrobacterium*‐mediated transient expression in *N. benthamiana* leaves. Constructs encoding full‐length BcGH61 (pCNG:BcGH61‐GFP), signal peptide‐deleted BcGH61 (pCNG:BcGH61^ΔSP^‐GFP), and GFP control (pCNG:GFP) were expressed in leaf tissues. Western blot analysis of total protein extracts, apoplastic fluid, and non‐secreted protein fractions showed that GFP‐tagged BcGH61 accumulated in both total and non‐secreted fractions from leaves expressing either full‐length BcGH61 or BcGH61^ΔSP^. In contrast, apoplastic accumulation was specific to leaves expressing the full‐length protein (Figure S1a). Confocal microscopy at 48 h post‐infiltration revealed distinct localisation patterns: BcGH61‐GFP fluorescence accumulated in the apoplastic and cytoplasmic space, while both BcGH61^ΔSP^‐GFP and free GFP localised to the cytoplasm and plasma membrane (Figure 1e). Collectively, these findings confirm that BcGH61 requires its native SP for apoplastic targeting and establishes its extracellular localisation in host plant cells following secretion from *B. cinerea* .

### 2.2 BcGH61 Triggers Severe Plant Cell Death in *N. benthamiana*

**FIGURE 2:**
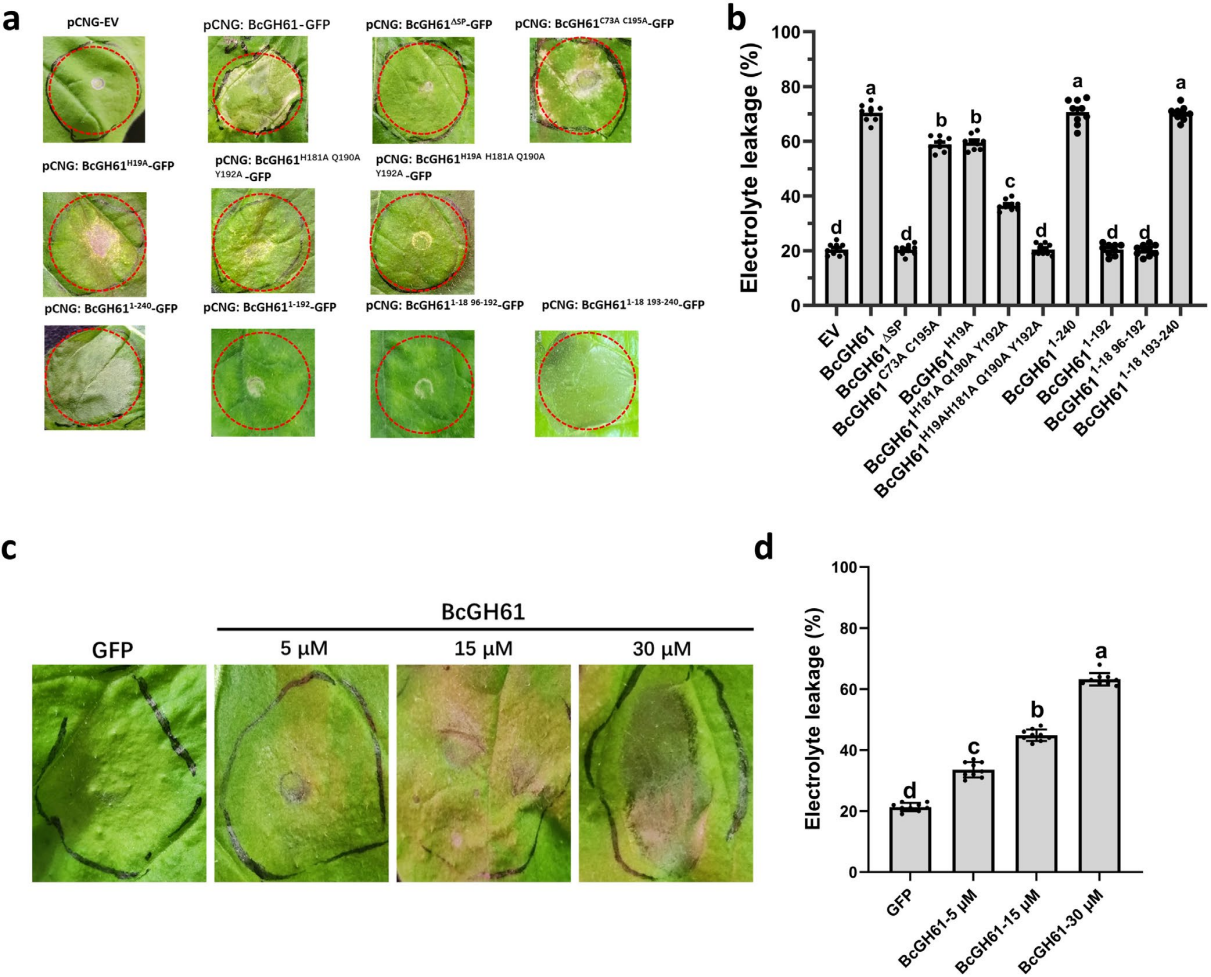
BcGH61 from *Botrytis cinerea* is a secreted elicitor of cell death in *Nicotiana benthamiana*. (a) *Agrobacterium* strains transformed with the indicated genes were infiltrated into leaves of *N. benthamiana*. The infiltrated areas were outlined with red dashed lines. 3 days after agroinfiltration, representative *N. benthamiana* leaves were taken. (b) Electrolyte leakage was assessed to quantify cell death in *N. benthamiana* leaves infiltrated with the respective constructs. Leaf samples were collected at 3 days post‐infiltration (dpi), and the assay was performed following a standard protocol. Values represent the mean ± standard error (SE) of three biological replicates. (c, d) Infiltration assay of the purified BcGH61 protein on *N. benthamiana* leaves. BcGH61‐GFP fused recombinant protein with His tag were produced and purified from *Escherichia coli* . (c) Images were taken 2 days after infiltration of the *N. benthamiana* leaves with 5, 15, and 30 μM solution of the purified protein. (d) Cell death induced by various concentration of BcGH61‐GFP proteins was assessed by electrolyte leakage measurements, as in panel (b). In both (b) and (d), data are presented as mean ± SD (*n* = 9, combining three biological replicates with three technical replicates each). Different lowercase letters indicate statistically significant differences (*p* < 0.01) as determined by one‐way ANOVA.

To evaluate the plant cell death‐inducing activity of BcGH61, we transiently expressed full‐length BcGH61 (pCNG:BcGH61‐GFP), its SP‐deleted variant (pCNG:BcGH61^ΔSP^‐GFP), and GFP control (pCNG:GFP) in *N. benthamiana* leaves. Distinct cell death symptom developed in leaves expressing full‐length BcGH61 within 72 h post‐infiltration, while BcGH61^ΔSP^ and GFP controls showed no visible symptoms (Figure 2a,b). Immunoblot analysis using anti‐GFP antibodies confirmed comparable expression levels of all constructs (Figure S1b), demonstrating that the SP guides BcGH61 secreted into the leaf apoplastic space to induce cell death. To determine whether extracellular BcGH61 alone could elicit cell death, we heterologously expressed His‐tagged BcGH61 in *Escherichia coli* Rosetta‐gami (DE3), with free GFP serving as a negative control. Coomassie Brilliant Blue staining confirmed successful protein induction (Figure S1c), followed by nickel‐affinity purification. Infiltration of 30 μM BcGH61 into *N. benthamiana* leaves induced rapid tissue collapse within 48 h, significantly exceeding the minimal response observed in GFP‐treated controls. A concentration‐dependent effect was evident, with 15 and 5 μM treatments showing progressively reduced but still significant cell death compared to controls (Figure 2c,d). Thus, these results confirmed the plant cell death‐inducing activity of BcGH61.

### 2.3 BcGH61 Requires Catalytic Residues for Full Phytotoxic Activity

Structural homology modelling and Consurf conservation analysis identified a conserved catalytic pocket harbouring four residues (H19, H181, Q190, Y192) critical for the enzymatic activity (Figure S2a–c). Bioinformatic analysis revealed that BcGH61 homologues are phylogenetically restricted to fungal saprotrophs and pathogens (Figure S2d). To test whether enzymatic function drives cell death induction, we generated catalytically deficient BcGH61 variants through site‐directed mutagenesis, including single (BcGH61^H19A^), triple (BcGH61^H181A Q190A Y192A^), and quadruple (BcGH61^H19A H181A Q190A Y192A^) substitutions. Transient expression of all mutants in *N. benthamiana* via *Agrobacterium* infiltration resulted in attenuated cell death phenotypes compared to wild‐type BcGH61, while single and triple point‐mutated proteins maintained the ability to induce leaf cell death, the quadruple mutant demonstrated complete loss of its phytotoxic activity (Figure 2a,b). Immunoblot analysis confirmed comparable protein accumulation across all constructs (Figure S1b), excluding expression‐level artefacts. Thus, these findings demonstrate that the full cell death‐inducing activity of BcGH61 is dependent of its catalytic activity.

### 2.4 Structural Disulphide Bonds Mediated by Conserved Cysteines Are Essential for BcGH61 Phytotoxicity

Conserved cysteine residues often stabilise protein tertiary structure through disulphide bonding, a critical feature for effector function in phytopathogens (Sevier and Kaiser 2002; Stergiopoulos and De Wit 2009). Structural modelling and Consurf evolutionary conservation analysis identified two buried cysteine residues (C73 and C195) forming a predicted intramolecular disulphide bond within the BcGH61 catalytic domain. These residues are invariant across fungal GH61 homologues, suggesting essential structural roles (Figure S2a,b). To further clarify the role of BcGH61 conserved cysteine residues for its cell death‐inducing activity, site‐directed mutagenesis was conducted to replace cysteine residues with alanine (C73A and C195A). Transient expression in *N. benthamiana* revealed severe attenuation of cell death induction in BcGH61^C73A C195A^ mutant, compared to native BcGH61 (Figure 2a,b), despite being efficiently expressed (Figure S1b). Thus, these results demonstrate that the C73‐C195 disulphide bridge is critical for the cell death‐inducing activity of BcGH61.

### 2.5 Identification of BcGH61 Functional Domains Essential for Plant Cell Death Induction

To determine the critical region of BcGH61 responsible for cell death induction, we generated a series of truncated variants based on Consurf‐predicted conserved domains (Figure S2a,b), BcGH61^1–240^, BcGH61^1–192^ and BcGH61^1–18 96–192^ were expressed in *N. benthamiana* using *A. tumefaciens* infiltration assay. While BcGH61^1–240^ retained full cell death‐inducing activity, both BcGH61^1–192^ and BcGH61^1–18 96–192^ completely lost this capacity. Thus, the BcGH61^1–18,193–240^ was selected to further test whether the specificity of this region leads to cell death. The result indicated that transient expression of BcGH61^1–18,193–240^ caused death of *N. benthamiana* leaves (Figure 2a,b). Immunoblot analysis demonstrated that all truncated proteins were expressed well in *N. benthamiana* leaves (Figure S1b). Hence, these findings demonstrate that the 193–240 amino acid segment of BcGH61 contains essential determinants for phytotoxic activity.

### 2.6 BcGH61 Activates Immune‐Associated Transcriptional Programmes in *N. benthamiana*

**FIGURE 3:**
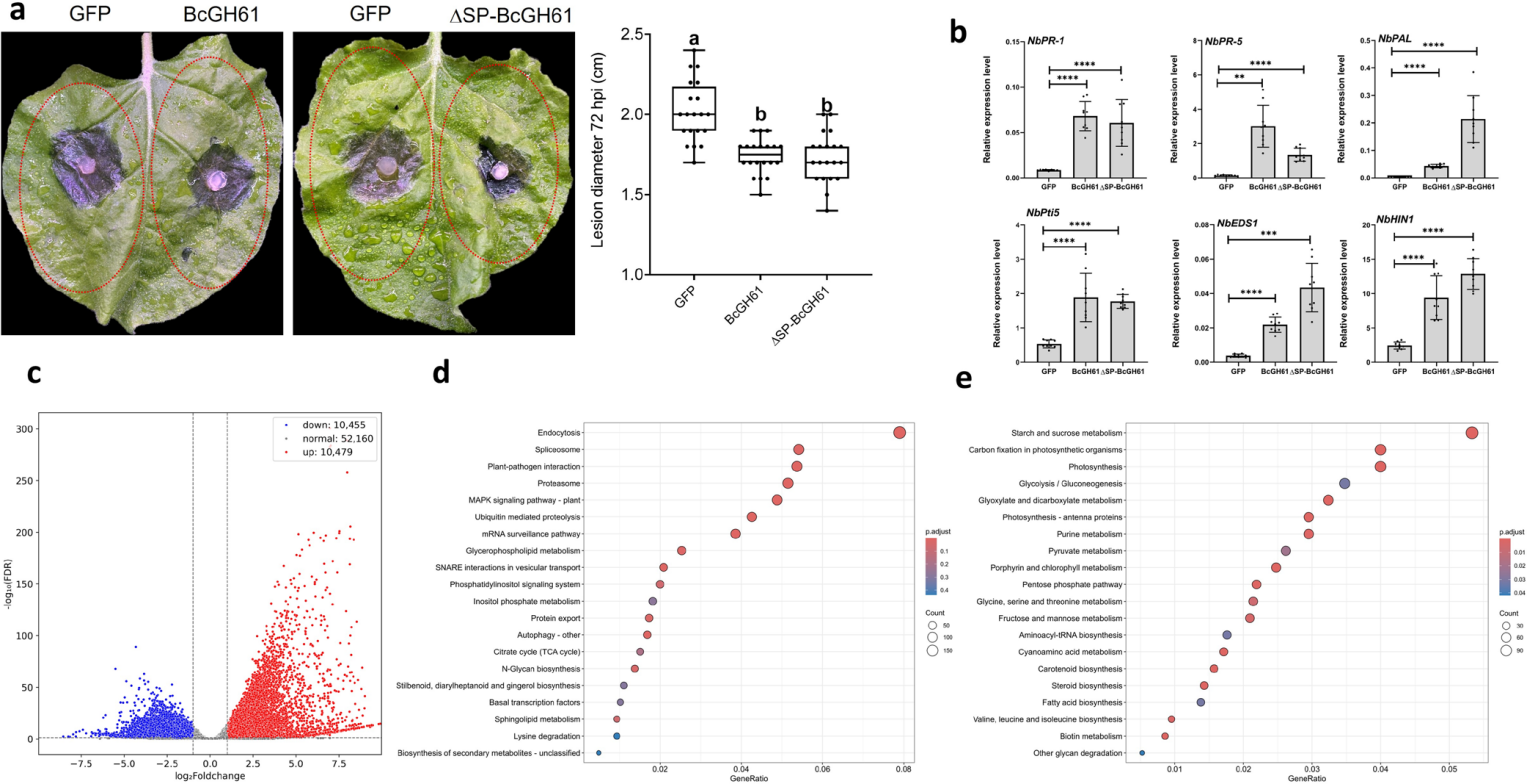
BcGH61 activates plant immunity and confers resistance against *Botrytis cinerea* infection. Leaves of *Nicotiana benthamiana* were infiltrated with *Agrobacterium* strains that harboured construct encoding GFP (35S:GFP), BcGH61 (35S:BcGH61), or ΔSP‐BcGH61 (35S:ΔSP‐BcGH61). (a) Infection assay of *B. cinerea* . Two days after agroinfiltration, the leaves were challenged with *B. cinerea* mycelial plugs, the inoculated plants were incubated for an additional 72 h in a moist chamber and then disease progression was documented. The area outlined by the red dashed line indicates the site of agroinfiltration. All data from three independent biological replications are indicated as black dots. Whiskers of the boxplots show the minimum and maximum values; centre lines of boxplots display the median values; box limits indicate the 25th and 75th percentiles. Different lowercase letters denote statistically significant differences at *p* ≤ 0.01 using one‐way ANOVA. (b) The relative expression of defence‐related marker genes in *N. benthamiana* that transiently expressed indicated constructs. Samples were harvested 2 days after agroinfiltration, then gene expression levels were determined by reverse transcription‐quantitative PCR and normalised with *EF‐1α* gene of *N. benthamiana*. Expression in GFP treatment plants was set as control. Data represent mean ± SD (*n* = 9) from three independent biological replicates with three technical replicates. Asterisks represent significant differences of all other treatments compared to GFP treatment control (****p < 0.0001, ***p < 0.001, **p < 0.01, one‐way ANOVA). (c) Global transcriptome reprogramming induced by BcGH61. Volcano plot showing upregulated (red) and downregulated (blue) genes in the BcGH61‐expressing leaf samples compared with that in the GFP‐expressing (Control) leaf samples. (d, e) KEGG enrichment analysis of upregulated (d) and downregulated (e) differentially expressed genes from BcGH61‐ versus GFP‐expressing tissues. Circle sizes indicate gene counts per pathway, colour gradients represent adjusted *p*‐values. The top 20 pathways with the most significant *p*_adj_ values are shown.

Recent studies have shown that certain CDIPs of *B. cinerea* can be detected by the plant immune system, triggering defence responses (Bi et al. 2023). So, we investigated BcGH61‐triggered immune responses and disease resistance against *B. cinerea*; constructs encoding pCNG:BcGH61‐GFP, pCNG:BcGH61^ΔSP^‐GFP and pCNG:GFP were agroinfiltrated into *N. benthamiana*. Leaves transiently expressing these proteins were further challenged with fungal mycelial plugs at 2 days agroinfiltration. The results revealed that both BcGH61‐ and BcGH61^ΔSP^‐expressing leaves exhibited enhanced resistance to *B. cinerea* compared with that displayed by GFP‐expressing leaves (Figure 3a). To examine whether BcGH61 triggers defence‐related gene expression, 2 days after agroinfiltration, reverse transcription‐quantitative PCR (RT‐qPCR) was used to analyse the transcriptional changes in genes associated with the salicylic acid (SA) signalling pathway (*NbEDS1*, *NbPR1*, *NbPR5*), hypersensitive response (*NbHR*) regulator (*NbHIN1*), PTI‐associated gene (*NbPIT5*) and phenylpropanoid biosynthesis enzyme (*NbPAL*). Full‐length BcGH61 and BcGH61^ΔSP^ proteins both induced transcriptional activation of these defence‐related genes compared to those in leaves expressing GFP (Figure 3b), suggesting that in addition to exhibiting phytotoxicity, BcGH61 can also stimulate defence responses of the host plant. Notably, this pattern contrasts with the SP‐dependent cell death phenotype observed in Figure 2a, revealing distinct mechanistic requirements for phytotoxicity and immune activation. Intriguingly, despite its complete loss of enzymatic activity, the quadruple mutant BcGH61^EM^ (Enzymatic activity Mutant of BcGH61) retained the ability to activate plant immune responses at levels comparable to the native BcGH61 protein (Figure S3). Thus, these findings demonstrate that the immunity‐elicitor activity of BcGH61 is independent of its catalytic activity.

A transcriptome‐scale investigation of the possible effects of BcGH61 on host plant was performed by RNA‐seq analysis comparing BcGH61‐expressing and GFP‐expressing *N. benthamiana* leaves. Differential expression analysis identified 20,934 differentially expressed genes (DEGs) (log_2_FC ≥ |1|, adjusted *p*‐value ≤ 0.05), with near‐equivalent numbers of upregulated (10,479) and downregulated (10,455) transcripts in BcGH61‐expressing plants compared to GFP control (Figure 3c, Table S1), indicating BcGH61 expression induces widespread transcriptional changes. KEGG enrichment analysis showed that genes associated with Endocytosis (ko04144), Plant–pathogen interaction (ko04626), MAPK signalling pathway‐plant (ko04016), SNARE interactions in vesicular transport (ko04130) were over‐represented among the DEGs upregulated in the BcGH61‐expressing line (Figure 3d, Table S2). The downregulated DEGs predominated in processes linked to Photosynthesis (ko00195), Starch and sucrose metabolism (ko00500), Carbon fixation in photosynthetic organisms (ko00710) and Glyoxylate and dicarboxylate metabolism (ko00630) (Figure 3e, Table S3). These findings strongly demonstrate BcGH61's capacity to simultaneously activate plant immune responses (particularly MAPK signalling and pathogen recognition systems) and suppress photosynthetic machinery/central metabolism. The coordinated induction of endocytic trafficking and SNARE‐mediated processes suggests potential manipulation of host membrane dynamics, while metabolic downregulation aligns with characteristic pathogen‐induced resource reallocation strategies. These findings support BcGH61's role as a virulence effector that reprograms host physiology to favour infection progression.

### 2.7 BcGH61‐Induced Cell Death Operates Independently of NbBAK1/NbSOBIR1 Signalling

As a secreted effector, BcGH61 may potentially activate immune signalling through interactions with plant membrane receptor‐like kinases (RLKs) or receptor‐like proteins (RLPs), analogous to characterised CDIPs (Bi et al. 2023). To evaluate the involvement of the canonical coreceptor complex BAK1/SOBIR1 in this process, we employed tobacco rattle virus (TRV)‐mediated virus‐induced gene silencing (VIGS) to knock down *NbBAK1* and *NbSOBIR1* expression in *N. benthamiana*. Three weeks post‐VIGS treatment, RT‐qPCR confirmed substantial transcript reduction in target genes (Figure S4a). *Agrobacterium* containing the BcGH61 expression plasmid was subsequently agroinfiltrated into *GFP‐*, *NbBAK1‐* and *NbSOBIR1*‐silenced leaves, revealing comparable cell death phenotypes across all treatments (Figure S4b). Immunoblot analysis verified equivalent protein accumulation of BcGH61 in TRV:*GFP*, TRV:*NbBAK1*, and TRV:*NbSOBIR1* plants (Figure S4c). These results demonstrate conclusively that BcGH61‐triggered cell death bypasses the BAK1/SOBIR1 signalling module, suggesting either direct phytotoxic activity or engagement of alternative unidentified RLK or RLP.

### 2.8 BcGH61 Contributes to the Full Virulence of *B. cinerea*

**FIGURE 4:**
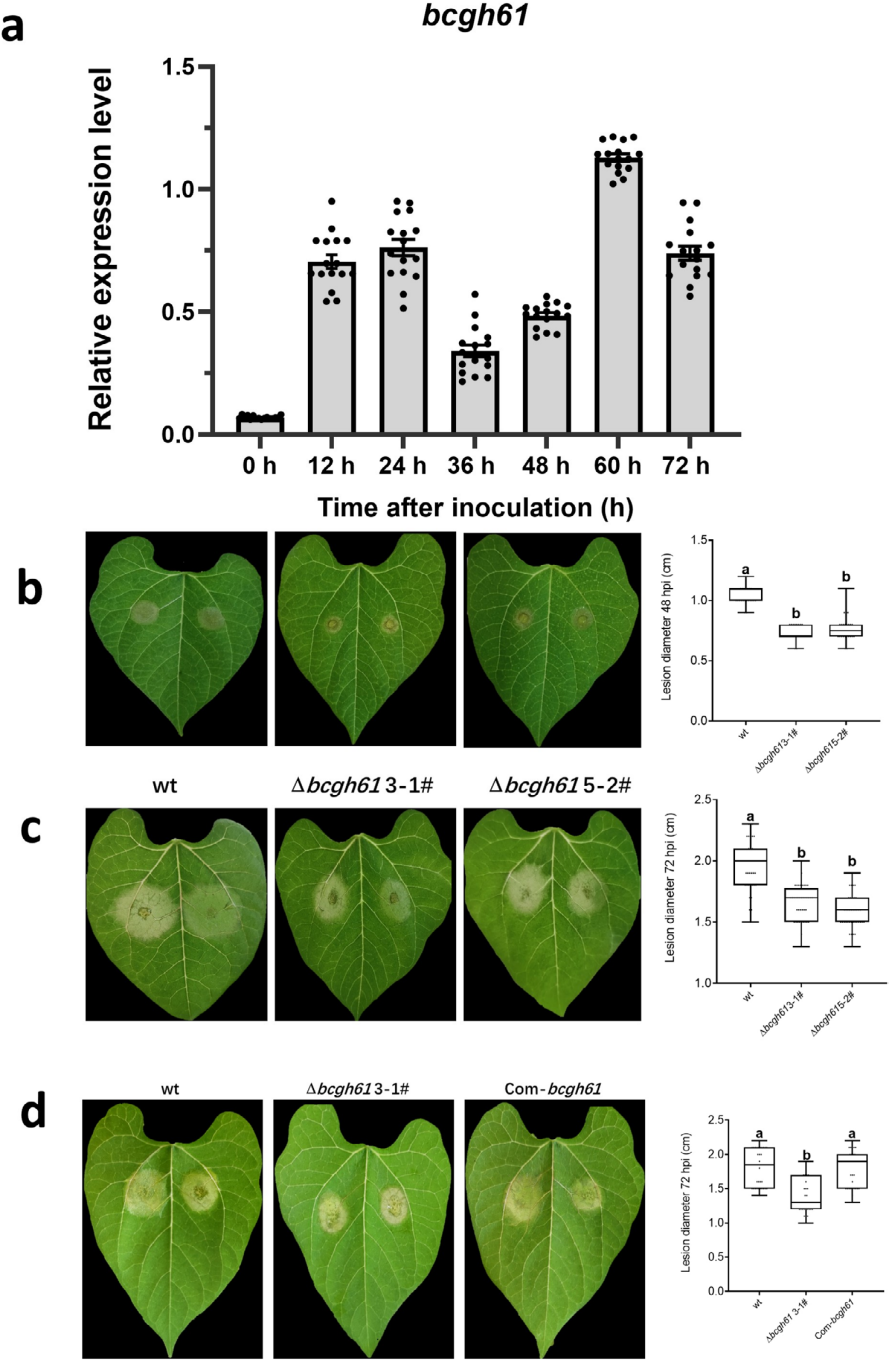
BcGH61 is a crucial virulence effector of *Botrytis cinerea*. (a) Reverse transcription‐quantitative PCR (RT‐qPCR) analysis of *bcgh61* during infection stages. The expression pattern of *bcgh61* was assessed by RT‐qPCR using *bcgpdh* as a reference gene for normalisation. Transcript levels were quantified using the comparative *C*_t_ method, with the expression level of *bcgh61* at 0 h on French bean leaves set as the baseline control. Data represent mean ± SD (*n* = 16) derived from four independent biological replicates, each with four technical replicates. (b–d) Infection assay of *B. cinerea* strains. Bean leaves were inoculated with spore suspensions of *B. cinerea* wild type strain (wt), *bcgh61* knockout strain (Δ*bcgh61*), *bcgh61* complemented strain (Com‐*bcgh61*). Lesion phenotypes were photographed and recorded at 48 h post‐inoculation(hpi) (b) and 72 hpi (c, d, respectively). Boxplots depict lesion sizes, where box limits represent the 25th and 75th percentiles, central lines indicate medians, and whiskers extend to the minimum and maximum values. Individual data points are shown as black dots. Data were pooled from at least 31 lesions across three independent biological replicates. Statistical significance (one‐way ANOVA, *p* ≤ 0.01) is denoted by distinct lowercase letters above groups.

To clarify the expression pattern of *bcgh61* during plant infection, bean leaves were inoculated with *B. cinerea* spores. RT‐qPCR analysis revealed that *bcgh61* transcript levels were upregulated during the early infection stage (0–24 h post‐inoculation [hpi]) (Figure 4a), potentially reflecting its role in establishing necrotic tissue regions. A transient downregulation of *bcgh61* expression was observed at the intermediate infection stage (36–48 hpi) (Figure 4a), followed by a sharp increase at 60 hpi, reaching a peak of approximately 16‐fold induction compared to baseline levels (0 hpi) (Figure 4a). Thus, this dynamic expression profile suggests that *bcgh61* plays a critical role in the progression of *B. cinerea* infection.

To further explore the role of BcGH61 in fungal pathogenicity and development, *bcgh61* deletion mutants (Δ*bcgh61*) and complemented strains (Com‐*bcgh61*) were generated and verified by PCR (Figure S5). Pathogenicity assays demonstrated that both Δ*bcgh61* mutants produced significantly smaller lesions on bean leaves at 48 and 72 hpi compared to the wild‐type (wt) strain (Figure 4b,c). The Com‐*bcgh61* strain restored virulence to wt levels, confirming that the attenuated pathogenicity was specifically linked to *bcgh61* disruption (Figure 4d). Notably, Δ*bcgh61* strains exhibited no detectable defects in colony morphology or growth rate on potato dextrose agar (PDA) (Figure S6), nor did they show altered sensitivity to cell wall stressors, including 0.5 mg/mL Congo Red (CR), 0.02% SDS, and 1M NaCl (Figure S6). GH61 proteins are known to mediate oxidative cleavage of crystalline cellulose in a copper‐ and reductant‐dependent manner (Lo Leggio et al. 2012). While BcGH61 significantly contributes to the full virulence of *B. cinerea* , our findings indicate that it is dispensable for hyphal growth and cell wall stress tolerance, highlighting its specific role in host tissue colonisation during infection.

### 2.9 BcGH61 Interacts With NbHrBP1 (Harpin Binding Protein 1) in *N. benthamiana*

**FIGURE 5:**
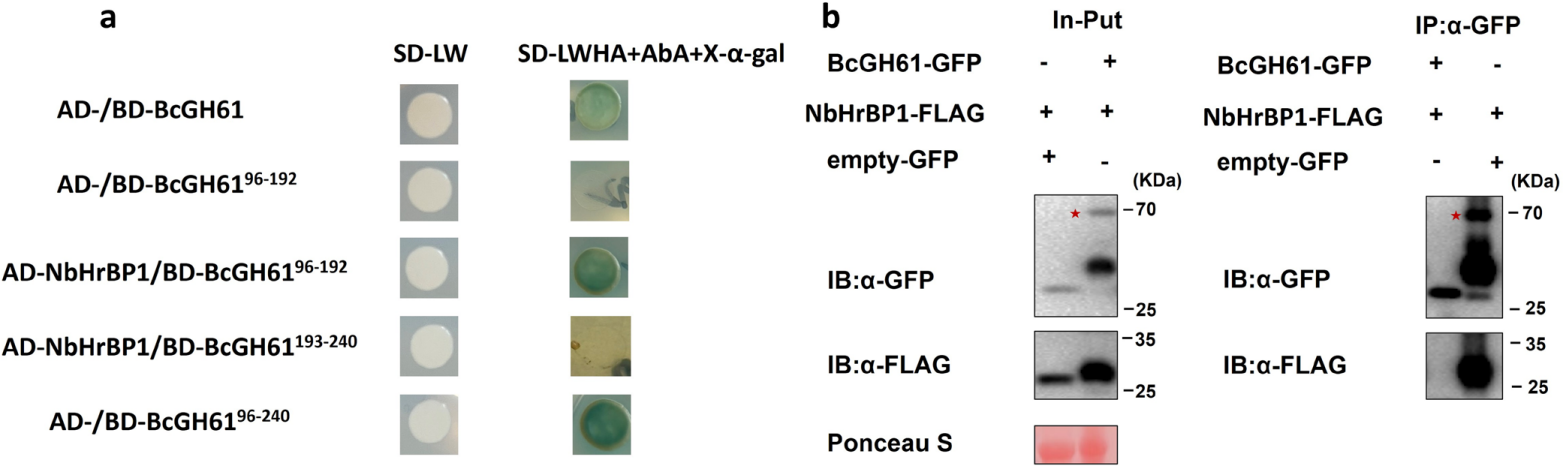
BcGH61 interacts with NbHrBP1 in *Nicotiana benthamiana*. (a) Yeast two‐hybrid assay validating the interaction between BcGH61 and NbHrBP1. SD/−Leu−Trp medium (SD−LW) was used to confirm successful plasmid co‐transformation. Positive protein–protein interactions were screened on SD/−Leu−Trp−His−Ade medium (SD−LWHA) containing aureobasidin A (AbA) and X‐α‐gal. (b) Co‐immunoprecipitation assay confirming the BcGH61‐NbHrBP1 interaction. *Agrobacterium*‐mediated transient expression was performed by co‐infiltrating *N. benthamiana* leaves with either BcGH61‐GFP or empty‐GFP vector control alongside NbHrBP1‐FLAG. Total proteins were immunoprecipitated with α‐GFP antibody (α‐GFP IP), followed by immunoblotting analysis using α‐FLAG antibody. NbHrBP1‐FLAG was specifically detected in complexes precipitated from BcGH61‐GFP samples but not from empty‐GFP controls.

**FIGURE 6:**
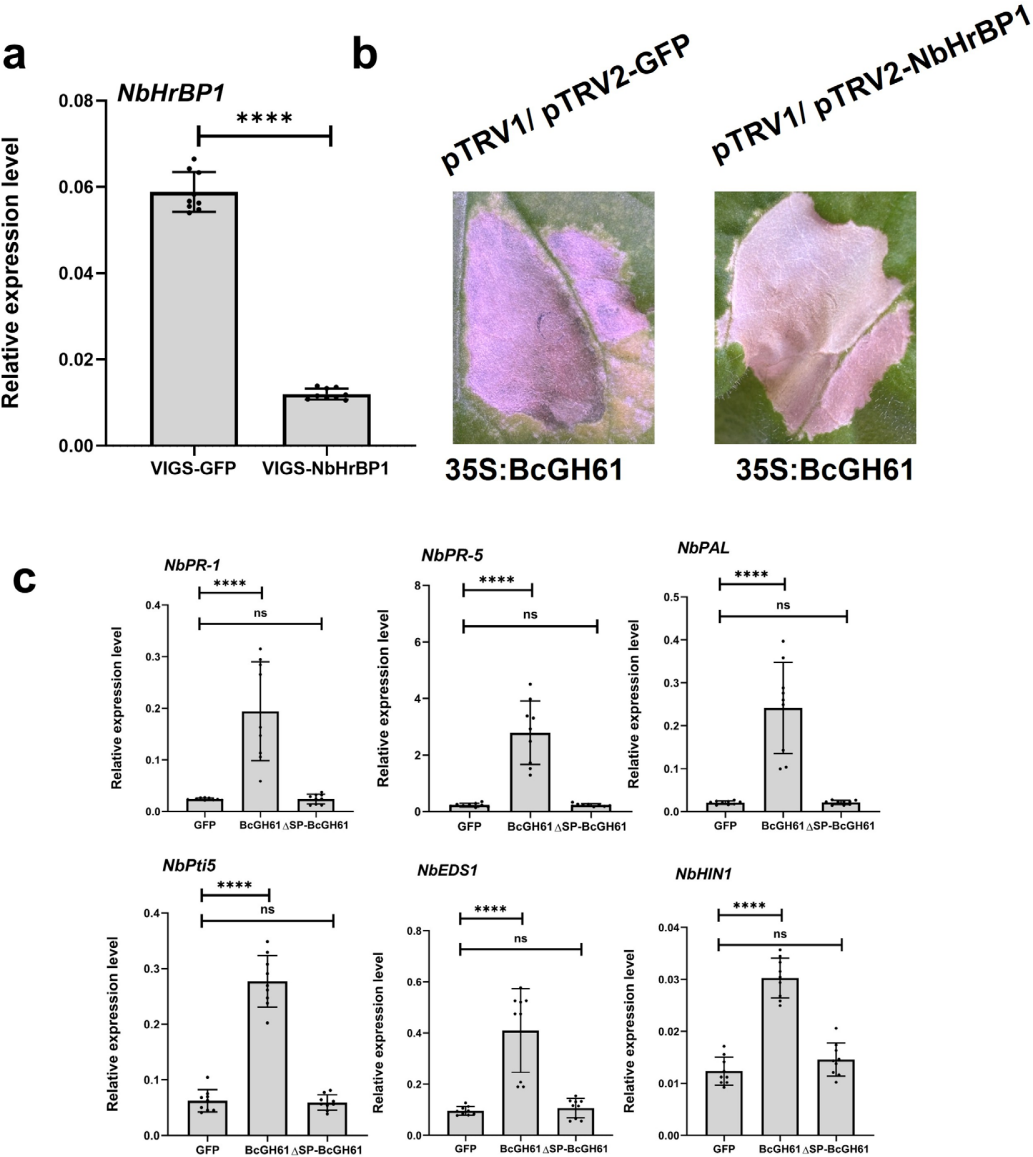
NbHrBP1 is dispensable for BcGH61‐induced cell death but required for intracellular immune perception in *Nicotiana benthamiana*. (a) Silencing efficiency validation of *NbHrBP1* by reverse transcription‐quantitative PCR (RT‐qPCR). The expression level of *NbHrBP1* in silenced plants (VIGS‐NbHrBP1) was measured. Data represent means ± SD from three biological replicates (each containing three technical replicates; *n* = 9). Asterisks denote statistically significant differences between VIGS‐NbHrBP1 and VIGS‐GFP controls (*p* < 0.0001, unpaired two‐tailed Student's *t* test). (b) BcGH61‐triggered cell death in *NbHrBP1*‐silenced plants. Virus‐induced gene silencing (VIGS) was performed using pTRV1/pTRV2‐NbHrBP1 constructs, with pTRV1/pTRV2‐GFP serving as the control. At 3 weeks post‐agroinfiltration, the 35S:BcGH61 recombinant protein was transiently expressed in silenced plants. Representative leaf phenotypes were documented at 5 days post‐infiltration (dpi). (c) Defence‐related gene expression analysis in *NbHrBP1*‐silenced plants. The relative expression of defence‐related marker genes in *N. benthamiana* that transiently expressed indicated constructs. Leaves were collected at 2 dpi after agroinfiltration, then gene expression levels were determined by RT‐qPCR and normalised with *EF‐1α* gene of *N. benthamiana*. Expression in GFP treatment plants was set as control. Data represent mean ± SD (*n* = 9) from three independent biological replicates with three technical replicates. Asterisks represent statistical differences of all other treatments compared to GFP treatment control (****p < 0.0001, ns *p* > 0.05, one‐way ANOVA).

To further dissect the immune response mechanism induced by BcGH61, we performed immunoprecipitation (IP) coupled with liquid chromatography–tandem mass spectrometry (LC–MS/MS) to identify plant proteins interacting with BcGH61. This analysis revealed a specific interaction between BcGH61 and *N. benthamiana* Harpin Binding Protein 1 (NbHrBP1) (Table S4). To confirm this interaction, yeast two‐hybrid (Y2H) assays were conducted. While full‐length BcGH61 and BcGH61^96–240^ displayed self‐activating activity in the Y2H system, a truncated variant lacking the autoactivation domain BcGH61^96–192^ successfully interacted with NbHrBP1 without inducing self‐activation (Figure 5a). The interaction was further validated in planta using co‐immunoprecipitation (Co‐IP) assays (Figure 5b). Notably, the BcGH61^193–240^ truncated variant harbouring key determinants required for phytotoxicity exhibits no detectable interaction with NbHrBP1 (Figure 5a). Further, we tested whether the BcGH61‐induced plant cell death was contributed by NbHrBP1. At 3 weeks after VIGS‐mediated *NbHrBP1* gene silencing, the silenced leaves were agroinfiltrated with BcGH61. As a result, BcGH61 did not lose its ability to induce cell death in the plants with the *NbHrBP1* gene silenced (Figure 6a,b). Interesting, we found the cytoplasmic‐localised BcGH61 (pCNG:BcGH61^ΔSP^‐GFP), but not the secreted BcGH61 (pCNG:BcGH61‐GFP), lost the activity of activation of the defence‐related genes in *NbHrBP1*‐silenced leaves (Figure 6c). These results position BcGH61 as a dual‐function effector that induces cell death via apoplastic localisation while simultaneously triggering intracellular immunity through NbHrBP1 perception. This functional decoupling suggests that phytotoxicity and host target recognition in BcGH61 may involve spatially distinct modular domains. Our findings suggest that NbHrBP1 may serve as a key recognition receptor for BcGH61, potentially enabling the detection of conserved fungal CDIPs and subsequent activation of defence‐related signalling pathways in *N. benthamiana*.

### 2.10 NbHrBP1 Participates in Host Defence Response to *B. cinerea* Infection

**FIGURE 7:**
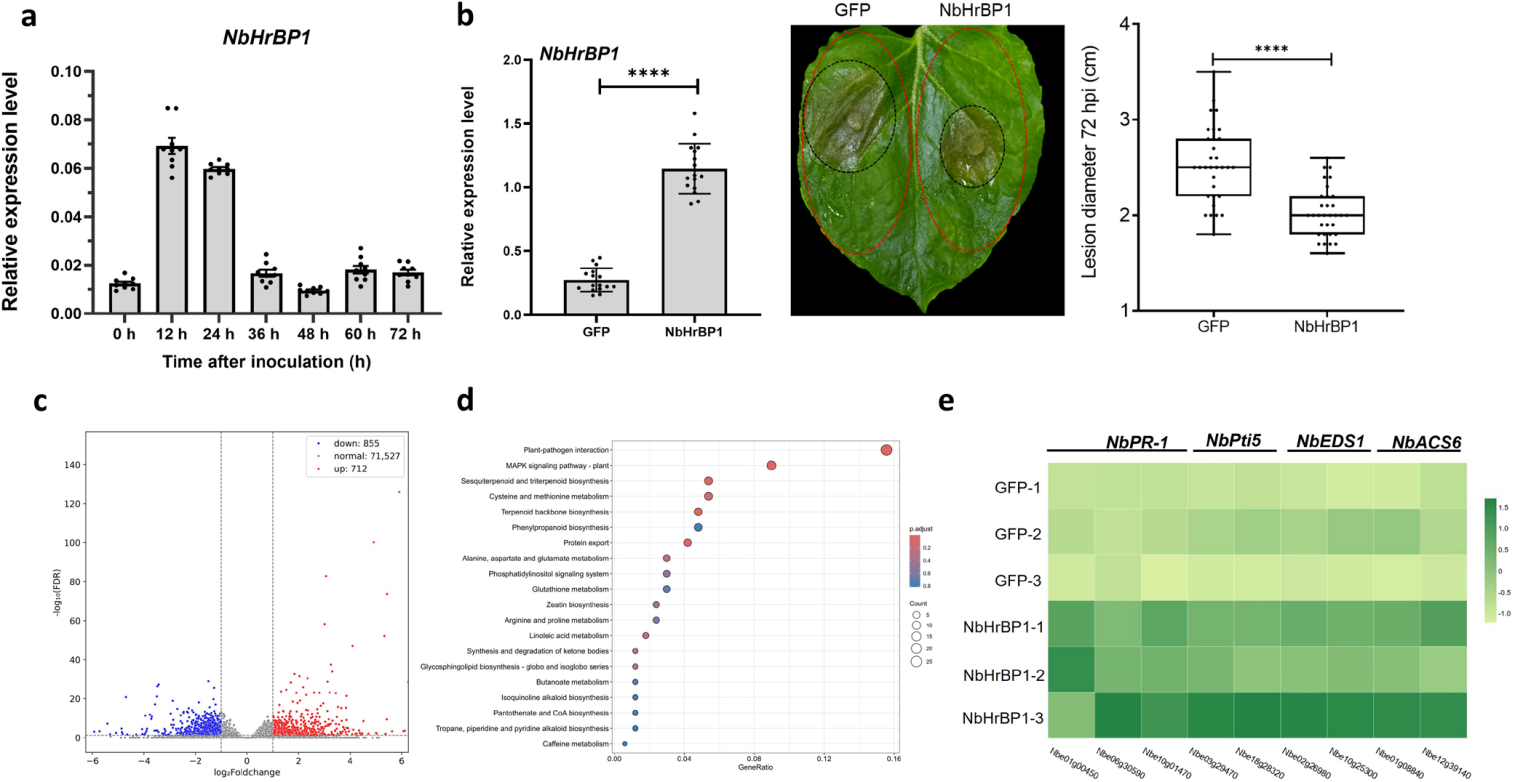
NbHrBP1 contributes *Nicotiana benthamiana* resistance to *Botrytis cinerea*. (a) Reverse transcription‐quantitative PCR (RT‐qPCR) analysis. The expression pattern of *NbHrBP1* during *B. cinerea* infection was analysed by RT‐qPCR, using *NbEF1α* as the reference gene for normalisation. Transcript levels were quantified using the comparative *C*_t_ method, with the expression level of *NbHrBP1* at 0 h serving as the baseline control. Data are presented as mean ± SD (*n* = 9), derived from three independent biological replicates, each with three technical replicates. (b) Phenotypic resistance assay. *NbHrBP1* overexpression (35S:NbHrBP1) and *GFP* control (35S:GFP) were transiently expressed in *N. benthamiana* via *Agrobacterium* infiltration. RT‐qPCR analysis was used for the validation of *NbHrBP1* overexpression. The red dashed contour demarcates the tissue sector subjected to agroinfiltration. Leaves were challenged with *B. cinerea* mycelia 48 h post‐infiltration. *NbHrBP1*‐overexpressing plants exhibited significantly reduced disease progression compared to *GFP* controls (****p < 0.0001, unpaired *t* test). Boxplots (median, quartiles, min/max) and biological replicates (black dots) confirm robust resistance conferred by overexpression of *NbHrBP1*. (c) Transcriptomic profiling. Volcano plots of differentially expressed genes (DEGs) between *NbHrBP1‐* and *GFP*‐transient expressing leaves. Red/blue dots represent upregulated/downregulated genes (log_2_FC ≥ |1|, adjusted *p* ≤ 0.05), and grey dots are nonsignificant. (d) KEGG enrichment analysis of upregulated DEGs. Circle size (gene count) and colour (adjusted *p*‐value) highlight significance. The top 20 pathways are shown. (e) Heatmaps showing the expression levels of genes encoding NbPR‐1, NbPti5, NbEDS1 and NbASC6. A colour bar is presented at the top right, and the colours from dark green to light green indicate high to low fold‐change (FC) values.

To clarify the expression pattern of *NbHrBP1* during plant infection, *N. benthamiana* leaves were inoculated with *B. cinerea* spores. RT‐qPCR analysis showed that *NbHrBP1* transcript levels were upregulated during the early infection stage (0–24 hpi), suggesting a potential role in the early host defence response. In contrast, *NbHrBP1* expression decreased sharply during the later infection stage (36–72 hpi) (Figure 7a). We further tested whether NbHrBP1 contributes to the host defence against *B. cinerea* . The results revealed that the constitutive overexpression of *NbHrBP1* attenuated the virulence of *B. cinerea* , with mild symptoms and smaller disease lesions than *GFP*‐expressing plants (Figure 7b). To investigate the molecular mechanisms underlying how NbHrBP1 participates in host defence response, RNA‐seq analysis was performed on *NbHrBP1*‐overexpressing and *GFP*‐expressing *N. benthamiana* leaves. The outcome was a set of 1576 DEGs in *NbHrBP1*‐overexpressing leaves, of which 712 DEGs were upregulated and 855 DEGs were downregulated (Figure 7c). The KEGG enrichment analysis showed that the upregulated DEGs were significantly enriched in Plant–pathogen interaction (ko04626), MAPK signalling pathway‐plant (ko04016) (Figure 7d). Among these are specific genes that are suggested to participate in the plant immune response, including receptors (e.g., CNCG: cyclic nucleotide gated channel), signalling proteins (e.g., calmodulin, CDPK, PTI5), and defence effectors (e.g., PRs, EDS1, HSP90, ASC6), and MAPK cascade components (e.g., MAPKKs, MPK4) (Figure 7e, Figure S7). These results support that NbHrBP1 acts as a positive regulator of immunity by reprogramming transcription to activate key defence pathways to *B. cinerea* .

## 3 Discussion

Beyond its fundamental roles in maintaining structural integrity and regulating development, the plant cell wall serves as a critical frontline barrier against abiotic stresses and invading pathogens. Comprising over 90% carbohydrates, primarily cellulose, hemicelluloses and pectic polysaccharides, the cell wall poses both a physical and biochemical challenge to pathogens (Popper et al. 2011; Rui and Dinneny 2020; Zhang et al. 2021). Hence, to colonise host tissues, most plant pathogens must degrade these polysaccharides. During colonisation, fungal and oomycete pathogens secrete a diverse array of GHs onto their cell surfaces and into the surrounding extracellular interaction zone. The repertoire of GHs varies significantly among plant‐associated microbes, reflecting adaptations to their specific ecological niches and lifestyles. In *B. cinerea* , GHs constitute the largest Gene Ontology (GO) category within its secretome, most of which target plant cell wall polymers, including cellulose, pectin and hemicellulose and other plant cell wall‐associated polysaccharides (Bi et al. 2021). Notably, BcCrh1 deviates from the canonical role of GHs in plant cell wall degradation. Instead, it participates in fungal cell wall biosynthesis by catalysing chitin‐glucan crosslinking. Intriguingly, BcCrh1 also functions as a cytoplasmic effector, eliciting plant cell death and activating defence responses—a dual role that underscores the complexity of pathogen–host interactions (Bi et al. 2021).

The GH61 family, currently reclassified as lytic polysaccharide monooxygenases (LPMOs) (Simmons et al. 2017), represents a unique class of cellulose‐degrading enzymes exhibiting dual catalytic activities involving both oxidative and hydrolytic mechanisms. While demonstrating weak endoglucanase activity, these copper‐dependent enzymes primarily facilitate lignocellulose degradation through oxidative cleavage of cellulose chains. This unique mechanism enables GH61 enzymes to disrupt the recalcitrant crystalline structure of cellulose substrates, thereby synergistically enhancing the performance of canonical cellulases in lignocellulosic biomass decomposition (Sun et al. 2021; Waghmare et al. 2021). Their structure is stabilised by disulphide bonds and features a conserved active site critical for substrate binding. Functionally, GH61 enzymes require transition metal ions (e.g., manganese or iron) as cofactors, with cellulase‐boosting activity dependent on the integrity of their metal‐binding site. Mutational studies highlight the importance of conserved residues in catalytic activity: substitutions of His1 to Asn and His68 to Ala abolished enzymatic function entirely, while Tyr153 to Phe mutations reduced activity. Notably, mutations in Gln151—which forms a hydrogen bond with Tyr153—resulted in severe activity loss, underscoring the essential role of the conserved H H‐X_8_‐Q/E‐X‐Y residues in GH61 proteins (Lo Leggio et al. 2012). Despite their well‐characterised role in polysaccharide modification, the contributions of GH61 proteins to phytopathogen virulence remain poorly understood. In this study, we identified BcGH61, a GH61 family protein secreted by *B. cinerea* , and demonstrated its essential role as a virulence factor during host infection. Notably, we confirmed the full cell death‐inducing capacity of BcGH61 strictly depends on its catalytic activity. This finding indicates that *B. cinerea* strategically employs BcGH61 to enzymatically dismantle plant cell wall polysaccharides during host colonisation. However, this degradative process risks releasing damage‐associated molecular patterns (DAMPs), such as oligogalacturonides, mixed‐linked glucans, xyloglucans and cellulose‐derived oligomers. These DAMPs are recognised by plant PRRs, triggering plant cell death and immune responses (Mélida et al. 2018; Rebaque et al. 2021).

**FIGURE 8:**
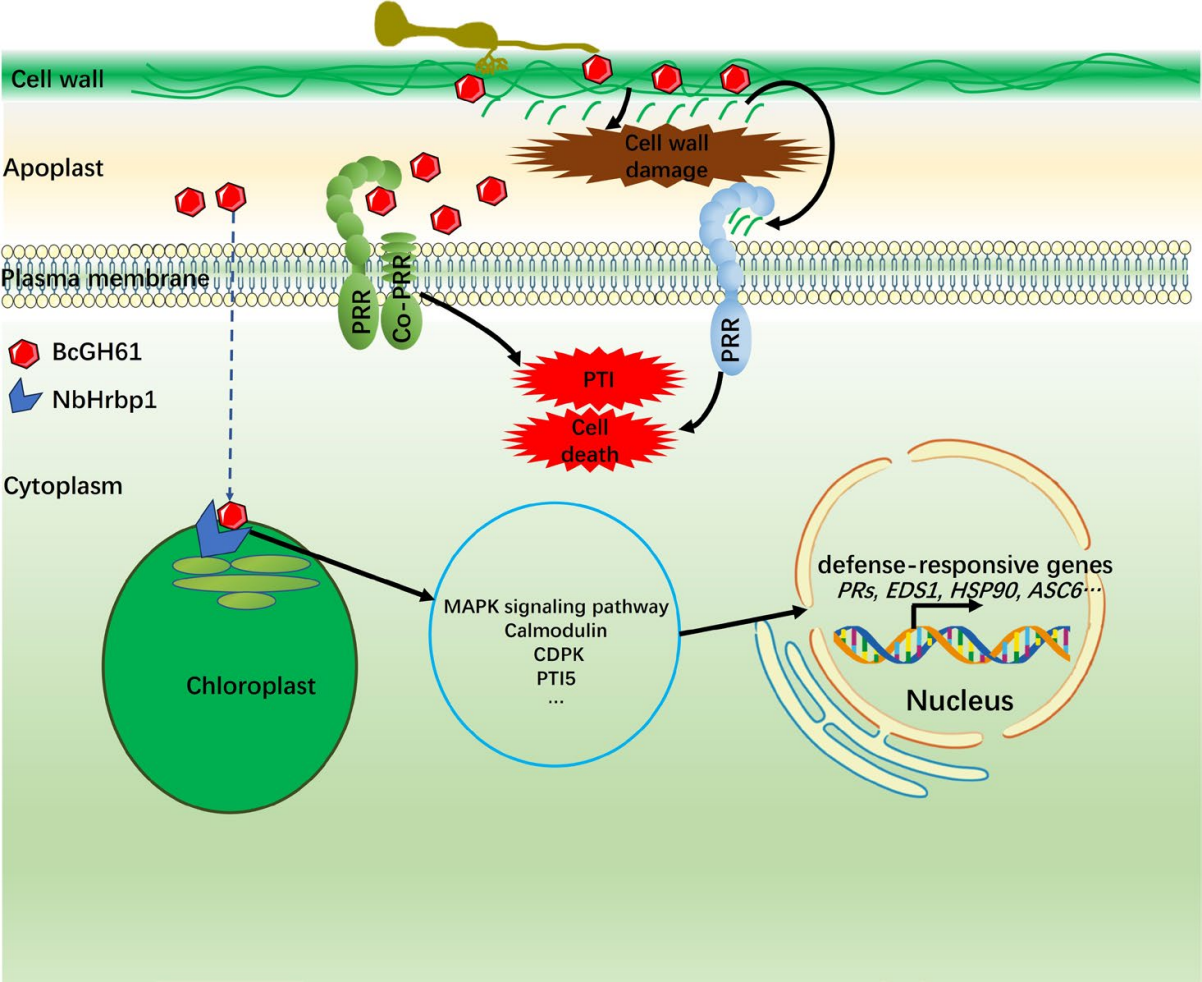
Working model of BcGH61‐mediated host colonisation by *Botrytis* *cinerea* . Following secretion into the apoplastic space, the virulence‐associated protein BcGH61 orchestrates plant cell death through dual extracellular pathways. Firstly, cell wall degradation‐mediated immunity activation: BcGH61 enhances cellulase activity to degrade plant cell wall components, thereby releasing damage‐associated molecular patterns (DAMPs). These DAMPs are subsequently recognised by plant pattern recognition receptors (PRRs), triggering both cell death and immune responses through classical defence signalling pathways. Alternatively, direct PRR engagement: BcGH61 may directly interact with unidentified PRRs or co‐receptor complexes (Co‐PRRs) to modulate pattern‐triggered immunity (PTI) or initiate phytotoxic signalling cascades. Simultaneously, BcGH61 employs an unidentified endocytic mechanism to enter plant cells and target chloroplasts. Within the chloroplast, it specifically localises to plastoglobules and thylakoid membranes by physically interacting with the host protein NbHrBP1. This intracellular activity activates a multi‐layered defence response involving: Phosphorylation cascades through MAPK signalling pathways, calcium‐mediated signalling via calmodulin networks, and transcriptional reprogramming of defence‐related genes. Collectively, these coordinated mechanisms paradoxically enhance plant immunity while promoting host cell death—a hallmark of *B. cinerea* infection. BcGH61 thus functions as a central virulence factor, manipulating both defence execution and cell death pathways to establish successful infection through its dual extracellular and intracellular actions.

Most characterised fungal CDIPs, including those from *B. cinerea* (except BcCrh1, BcCELP1), localise to the apoplast after secretion and require extracellular plant membrane components, typically involving the SOBIR1‐BAK1 receptor complex, to activate cell death (Kars et al. 2005; Zhu et al. 2017; Nie et al. 2019; Song et al. 2021). In this study, full‐length BcGH61 (containing its SP) induced severe cell death in *N. benthamiana*, whereas the SP‐truncated variant (BcGH61^ΔSP^) failed to elicit this response, indicating that BcGH61 causes cell death in the apoplast space (Figure 8). This conclusion was supported by further evidences, including yeast secretion assay and fluorescence observation of mesophyll cells following plasmolysis. It is noteworthy that while the complete cell death‐inducing capacity of BcGH61 depends on catalytic activity, the catalytically inactive BcGH61 mutant retains partial phytotoxic activity, suggesting the existence of additional signalling components beyond the canonical DAMPs‐PRR pathway. Notably, BcGH61‐triggered cell death, like that induced by *B. cinerea* BcHip1 (Jeblick et al. 2023) and *V. dahliae* VdEIX3 (Yin et al. 2021), operates independently of the canonical NbBAK1/NbSOBIR1 signalling axis. Intriguingly, the necrotrophic effector VmE02 from *Valsa mali* requires a host‐specific RLP in *N. benthamiana* for activity (Nie et al. 2019). This parallel suggests that BcGH61 may similarly cooperate with unidentified PRRs and/or Co‐PRRs to mediate PTI or phytotoxic signalling, potentially through a novel receptor complex (Figure 8).

Harpin‐binding protein 1 (HrBP1), a key plant defence regulator localised in plastoglobules and thylakoid membranes (Singh et al. 2010), was initially identified as the receptor mediating plant recognition of Harpin—a critical elicitor protein secreted by the fire blight pathogen *Erwinia amylovora* (Wei et al. 1992), this molecular interaction activates two major defence mechanisms: HR at infection sites and systemic acquired resistance (SAR) throughout the plant (El‐Maarouf et al. 2001; Gopalan 2008). Subsequent studies have revealed evolutionary conservation of HrBP1 orthologs across diverse plant species including *Arabidopsis thaliana* , soybean ( *Glycine max* ), tomato ( *Solanum lycopersicum* ). These orthologs maintain conserved structural features with molecular masses approximating 30 kDa (Iglesias‐Sanchez et al. 2023). Upon activation, HrBP1 selectively modulates key defence signalling pathways—including salicylic acid (SA), ethylene (ETH), and jasmonic acid (JA)—to orchestrate broad‐spectrum resistance against bacterial, viral, fungal and insect pathogens. For instance, HrBP1 upregulates *HIN1* (Harpin‐induced gene 1), a pathogenesis‐related gene that enhances mitogen‐activated protein kinase (MAPK) activity to amplify disease resistance (Lee et al. 2001). Similarly, the antiviral agent dufulin primes HrBP1‐mediated SA signalling, underscoring its role in chemically induced immunity to generate antiviral responses (Chen et al. 2012). In this study, we demonstrate that BcGH61, a secreted effector from *B. cinerea* , physically interacts with the single‐copy HrBP1 homologue in cytoplasmic space of *N. benthamiana*, suggesting the translocation of the BcGH61 protein into plant cells from the apoplastic space by deploying unidentified uptake mechanisms (Figure 8). This finding also raises critical questions about the evolutionary conservation of BcGH61‐HrBP1 interactions across host plants and their potential as targets for engineered resistance against *B. cinerea* . In this regard, future investigations into the biochemical dynamics of this interaction and its downstream signalling cascades will elucidate how pathogen‐derived effectors co‐opt plant receptors to subvert or activate immunity, offering novel strategies for crop protection.

Although most CDIPs from plant pathogens activate plant immune responses alongside host cell death, accumulating evidences suggest that cell death induction is not obligatory for triggering plant immunity. The N‐terminal domain of PevD1 is sufficient to elicit SAR, while its C‐terminal domain drives cell death. Crucially, SAR activation by PevD1 does not require its cell death‐inducing activity, indicating functional uncoupling of these processes (Liu et al. 2014). *Hyaloperonospora arabidopsidis* produces 10 Nep1‐like proteins (HaNLPs) that lack cell death‐inducing activity yet robustly activate immunity in *A. thaliana* (Oome et al. 2014). In *B. cinerea* , the cell death‐inducing activity of BcXYG1 depends on two critical peptide domains. Domain‐specific mutations abolish cell death induction but preserve its immune‐priming capacity, confirming separable signalling pathways (Zhu et al. 2017). Ps109281 from *P. sojae* triggers broad‐spectrum immune responses across plant species without eliciting cell death, further supporting the independence of immune activation from cytotoxicity (Wang et al. 2022). Collectively, these studies demonstrate that pathogen‐derived immune activators can engage plant defence mechanisms through cell death‐independent pathways. Here, our findings establish a dual‐action molecular paradigm for *B. cinerea* virulence, where BcGH61 orchestrates host colonisation through spatially partitioned mechanisms: extracellularly, it operates via apoplastic GH activity to degrade plant cell walls, generating DAMPs that activate PRR‐mediated cell death pathways and engaging canonical immune receptors through PTI or phytotoxic signalling cascades; intracellularly, it translocates into host cells via undetermined transport mechanisms and subsequently targets chloroplast compartments through physical interaction with NbHrBP1 at plastoglobule‐thylakoid interfaces. This interaction activates defence signalling nodes while paradoxically priming immunity‐related transcriptional reprogramming to generate necrotic microenvironments. The pathogen exploits these microenvironments as footholds for subsequent colonisation (Figure 8). It is now established that clathrin‐mediated endocytosis (CME) serves as a key pathway for the entry of cytoplasmic effectors from fungal and oomycete pathogens into plant cells. Future studies should focus on elucidating the molecular mechanism by which BcGH61 is secreted from the pathogen and enters host cells, which is crucial for understanding its complete molecular mechanism during infection (Wang et al. 2023). Notably, *NbHrBP1* transcript levels decreased sharply during later infection stages (36–72 hpi), following transient activation in early stages (0–24 hpi). This dynamic suggests that *B. cinerea* may employ undetermined immunosuppressive mechanisms to prevent excessive accumulation of *NbHrBP1*. This mechanistic dichotomy reveals an evolutionary arms race adaptation—the pathogen hijacks both damage perception and intracellular defence machinery to create a “controlled chaos” microenvironment favourable for invasion. Crucially, the decoupling of immune activation from direct tissue destruction (via intracellular NbHrBP1 engagement) suggests druggable targets for developing damage‐sparing immune potentiators that could enhance crop resilience without yield penalties. Future functional and phenotypic studies may provide insights into whether it can evade phytotoxic attacks to plant while maintaining its activated function in plant defence.

## 4 Experimental Procedures

### 4.1 Fungi, Bacteria, Plants and Culture Conditions

The *B. cinerea* wild‐type strain B05.10 and the derived transformants were grown and maintained on PDA (Acumedia) at 22°C under continuous fluorescent light supplemented with near‐UV (black) light. *B. cinerea* strains used in this study are listed in Table S5. The conidia of all *B. cinerea* strains were obtained after 7 days of culture. The *Escherichia coli* strains of DH5a and Rosetta‐gami (DE3) (Weidi) were respectively used for plasmid construction and protein expression. *A. tumefaciens* GV3101 (Pyeast) was used for *Agrobacterium*‐mediated transient expression of target proteins in plant leaves. *Saccharomyces cerevisiae* Y2HGold and YTK12 (Coolaber) were used for yeast two‐hybrid and signal sequence trap assay, respectively. *N. benthamiana* and bean ( *Phaseolus vulgaris* , genotype N9059) were grown in a greenhouse under 16 h/8 h intervals of light/dark at 25°C/22°C.

### 4.2 Vector Construction and Plant Transformation

Primers used in this study are listed in Table S6. The *bcgh61* deletion construct was generated as described previously (Zhu et al. 2017). The 5′ (500 bp) and 3′ (500 bp) flanking fragments of the *bcgh61* gene were amplified and cloned into the upstream and downstream regions, respectively, of the *hph* cassette using Gibson Assembly Master Mix kit (New England Biolabs). The complementary expression plasmid was generated by cloning the full‐length open reading frame of *bcgh61* into the pNAN‐OGG vector (carrying a nourseothricin resistance cassette) under the regulation of *Aspergillus nidulans* *oliC* promoter (NCBI identifier BN001307.1) and *B. cinerea* *tubA* terminator (NCBI identifier CP009817.1) as described previously (Bi et al. 2021).

For transient expression of the target full‐length protein and site mutant proteins in plants using the agroinfiltration method, the indicated sequences fused with the GFP tag were cloned into vector pCNG between the 2 × CaMV 35S promoter and the NOS terminator and then transformed into *A. tumefaciens* GV3101. Site‐directed mutagenesis products (BcGH61^H19A^, BcGH61^H181A Q190A Y192A^ BcGH61^H19A H181A Q190A Y192A^ and BcGH61^C73A C195A^) were generated using PCR and then were cloned into the pCNG. The *E. coli* protein expression vector was constructed by cloning the *bcgh61* mature sequence without the signal peptide (BcGH61^ΔSP^) into the vector pET14b (Beyotime) and then transformed into *E. coli* Rosetta‐gami (DE3).

To generate the constructs used for yeast two‐hybrid assay (Y2H), the ORFs of target genes without the secretion sequence were amplified using PCR, and the PCR amplification products were cloned into the pGADT7 and pGBKT7 yeast two‐hybrid vectors (Bi et al. 2021). To generate the vector used for yeast signal sequence trap assay, the 5′ (200 bp) fragment of *bcgh61* mature sequence was cloned into the pSUC2 T7M13ORI vector under the control of ADH1 promoter and β‐fructofuranosidase (Yin et al. 2018; Liu et al. 2024).

### 4.3 Bioinformatics Analysis

The genomic sequence database of *B. cinerea* (https://mycocosm.jgi.doe.gov/Botci1/Botci1.home.html) was used for BLAST searches of *B. cinerea* genes (Amselem et al. 2011). The SignalP 4.0 server (https://services.healthtech.dtu.dk/services/SignalP‐4.1/) was used to predict the presence of signal peptides and the location of their cleavage sites in the proteins (Teufel et al. 2022). TMHMM Server v. 2.0 (http://www.cbs.dtu.dk/services/TMHMM/) was used for the prediction of transmembrane helices in proteins (Krogh et al. 2001). The conserved protein domain search was performed by SMART MODE http://smart.emblheidelberg.de/smart/change_mode.pl (Letunic and Bork 2018), while the databases NCBI and UniProt were used for BLASTp searches. ConSurf (https://consurf.tau.ac.il/consurf_index.php) was used for evolutionary conservation analysis and functional domain prediction. Multiple Sequence Alignment (MSA) and Maximum Likelihood Tree (MLT) were performed by MEGA 11 and ClustalX to produce the pairwise alignment between the two proteins.

### 4.4 The Total Plant Protein Extraction and Western Blotting Assay

*Agrobacterium tumefaciens*‐mediated transient expression in *N. benthamiana* leaves was performed using the agroinfiltration method as previously described (Kettles et al. 2017). To extract the total protein of plants, approximately 0.2 g of tissue was ground to a powder using liquid nitrogen and suspended in 1 mL of cold lysis buffer (Beyotime). Then, the target samples were incubated on ice for 30 min and centrifuged at 13,000 *g* for 10 min at 4°C to collect the supernatant‐soluble proteins. The supernatant proteins were then mixed with 5 × SDS‐PAGE sample buffer (Beyotime) and denatured by boiling for 10 min at 100°C. The denatured proteins were separated by SDS‐PAGE electrophoresis and transferred onto PVDF membranes (0.45 mm). Western blotting was carried out using an anti‐GFP or anti‐FLAG antibody (Dia‐An).

### 4.5 Fluorescence and Confocal Microscopy

To analyse the subcellular localisation of BcGH61 in *N. benthamiana* cells, the BcGH61‐GFP, BcGH61^ΔSP^‐GFP, and GFP sequences were cloned into binary vector pCNG between the 2 × CaMV 35S promoter and NOS terminator. Samples were collected from *N. benthamiana* leaves 2 days after agroinfiltration. For plasmolysis, samples were infiltrated with 1 M NaCl solution for 30 min, then the samples were imaged under confocal laser scanning microscope (Leica, TCS SP8). GFP fluorescence was detected at an excitation wavelength of 488 nm and an emission wavelength of 495–510 nm. mCherry fluorescence was detected at 580 nm excitation and 579–650 nm emission.

### 4.6 Transformation, Pathogenicity and Cell Wall Stress Tolerance Assay of *B. cinerea*

*Botrytis cinerea* strains employed in this investigation are catalogued in Table S5. Genetic transformation of *B. cinerea* was performed as described previously (Ma et al. 2017). The *bcgh61* gene deletion mutants Δ*bcgh61* and complementation strains Com‐*bcgh61* were confirmed using PCR. Pathogenicity assays on the primary leaves of 9‐day‐old bean plants were performed as previously described (Zhu et al. 2017). Conidia of indicated *B. cinerea* strains were suspended in Gamborg's B5 medium (2% glucose and 10 mM KH_2_PO4/K_2_HPO_4_, pH 6.4), the conidia were diluted to 2 × 10^5^ conidia·mL^−1^ and leaves were inoculated with 7.5 μL of conidial suspension. Plants were incubated in a humid chamber at 22°C for 72 h, and the lesion diameter was measured. To determine the possible effect of BcGH61 on stress tolerance and cell wall integrity, the indicated strains were inoculated on PDA plates supplemented with 0.5 mg mL^−1^ Congo Red, 1 M NaCl, 20 mM H_2_O_2_ or 0.02% SDS at 22°C, as described previously (Zhu et al. 2017).

### 4.7 RNA Isolation and RT‐qPCR Analysis

Total plant or fungal RNA was isolated using the RN03‐RNApure Kit (Aidlab) according to the manufacturer's instructions and stored at −80°C. For cDNA synthesis, the HiScript III All‐in‐one RT SuperMix Kit (Vazyme) was used to remove DNA and generate the first‐strand cDNA. qPCR was performed using the CFX96 Touch Real‐Time PCR Detection System (Bio‐Rad) and SYBR Premix Ex Taq II (Takara) according to each manufacturer's instructions. The relative expression levels of the *B. cinerea bcgpdh* gene (*Bcin01g10320*) and the *N. benthamiana NbEF1a* gene were used as references for normalising the RNA sample. For examined genes, relative expression levels were determined using the 2^−ΔΔ*Ct*^ method with three independent biological replicates. Primers used in RT‐qPCR are listed in Table S6.

### 4.8 Purified Proteins Infiltration Assay on Leaves

Expression of BcGH61 and GFP recombinant proteins were performed in *E. coli* Rosetta‐gami (DE3) as described previously (Zhu et al. 2017). Purification of recombinant proteins was performed using glutathione beads 4FF (Smart‐Lifesciences) according to the manufacturer's instructions. The proteins were cleaned using Amicon Ultra‐4 centrifugal filter devices (Merck Millipore) to remove the elution buffer, dissolved in phosphate‐buffered saline and stored at −80°C. To test the phytotoxic activity of BcGH61, leaves were infiltrated with recombinant protein solution. Plants were then kept in chamber at 25°C and photographed at 2 or 3 days after treatment.

### 4.9 Induction of Plant Resistance by BcGH61

To test the induction activity of BcGH61 on plant defence responses and sensitivity to infection, *N. benthamiana* leaves were agroinfiltrated with vectors, including pCNG:BcGH61‐GFP, pCNG:BcGH61^ΔSP^‐GFP and negative control pCNG:GFP. The infiltrated plants were kept in a greenhouse for 48 h, after which the treated leaves were used to measure the expression levels of defence‐related marker genes using RT‐qPCR.

### 4.10 RNA‐seq Analysis

Transcriptome profiling was performed on *N. benthamiana* leaves collected 2 days post‐agroinfiltration. Two experimental groups were analysed: (1) transiently expressed free GFP control plants and (2) BcGH61‐expressing plants. Leaf tissues were immediately flash‐frozen in liquid nitrogen following collection. Total RNA extraction was conducted using the Spin Column Plant Total RNA Purification Kit (Sangon Biotech) according to the manufacturer's specifications. RNA‐seq services were provided by Wuhan Gene Read Biotechnology Co. Ltd., Wuhan, China (www.genereadtech.com/). The cDNA libraries were constructed and sequenced on the Illumina HiSeq platform (Illumina Inc.). Raw RNA‐Seq data were processed using fastp v. 0.23.2 to remove adaptor sequences and low‐quality reads. Quality‐filtered reads were then aligned to the *N. benthamiana* reference genome (https://bioweb01.qut.edu.au/benthTPM/download.html) using HISAT2 v. 2.2.1 with default parameters. Gene expression quantification was performed using featureCounts v. 2.0.1, with transcript abundance normalised as fragments per kilobase of transcript per million mapped reads (FPKM). Differential expression analysis between GFP‐ (control) and BcGH61‐ or NbHrbp1‐agroinfiltrated groups was conducted using DESeq2 v. 1.20 with three biological replicates. DEGs were identified using cut‐off criteria of the log_2_fold‐change ≥ ||1 and FDR (false discovery rate) < 0.05. The resulting DEGs were subsequently analysed for functional enrichment through GO terms and Kyoto Encyclopedia of Genes and Genomes (KEGG) pathways using clusterProfiler v. ‐4.2.0. Gene Set Enrichment Analysis (GSEA) was implemented to detect statistically over‐represented biological processes and pathways.

### 4.11 Co‐Immunoprecipitation Assay

For the co‐immunoprecipitation assay, fusion vectors were introduced into *A. tumefaciens* GV3101. These cells were then infiltrated into *N. benthamiana* leaves, resulting in the expression of BcGH61‐GFP and GFP proteins. After 2 days, the agroinfiltrated leaves were collected and total protein extraction was performed using IP lysis buffer (Beyotime) containing 1 mM phenylmethanesulfonyl fluoride (PMSF). The mixture was centrifuged at 13,000 *g* for 15 min at 4°C, and the supernatant was subsequently incubated with 30 μL GFP‐nanoab‐agarose beads (ChromoTek) at 4°C for 16 h. The beads were subjected to three washes using 500 μL of pre‐cooled IP wash buffer. Subsequently, the samples were subjected to SDS‐PAGE, after which the corresponding strips were excised and decoloured. Following this, the samples were subjected to trypsin digestion, and the resulting processed samples were analysed by liquid chromatography‐mass spectrometry (LC–MS/MS) in order to obtain the raw mass spectrometry results. These results were analysed by the software MaxQuant v. 1.6.2.10 to match the data and obtain the identification results.

To verify the interaction between BcGH61 and host plant proteins, BcGH61‐GFP, GFP, and host plant proteins tagged with FLAG (pCNF3) were transiently co‐expressed through agroinfiltration in the leaves of 4‐ to 5‐week‐old *N. benthamiana* leaves. Total proteins were extracted using a cell lysis buffer, and then incubated with GFP‐nanoab‐agarose beads as previously described. The eluted proteins were analysed by immunoblotting using anti‐GFP and anti‐FLAG antibodies, with total proteins loaded as an input control.

### 4.12 Liquid Chromatography‐Mass Spectrometry

Ultraperformance liquid chromatography/mass spectrometry‐grade solvents were used for all chromatographic steps. Each sample was loaded using a splitless nano‐ultra‐performance liquid chromatograph (10 kpsi nanoAcquity; Waters). The mobile phase was water + 0.1% formic acid (A) and acetonitrile + 0.1% formic acid (B). Desalting of the samples was performed online using a reverse‐phase C18 trapping column (150 μm i.d., 5 cm length, 3 μm particle size; Waters). The peptides were then separated using a C18‐AQ nanocolumn (100 μm i.d., 180 mm length, 3 μm particle size; Waters) at 600 nL min^−1^. Peptides were eluted from the column into the mass spectrometer using the following gradient: from 4% to 8% B in 2 min, from 8% to 28% B in 33 min, from 28% to 40% B in 20 min, from 40% to 95% B in 1 min and from 95% to 95% B in 10 min, and then back to the initial conditions.

### 4.13 Yeast Two‐Hybrid Assay and Yeast Signal Sequence Trap Assay

To verify proteins that interact with BcGH61 in *N. benthamiana*, a yeast two‐hybrid screen was performed using the Matchmaker GAL4‐based two‐hybrid system. The BcGH61^ΔSP^ protein was inserted into the yeast bait vector pGBKT7, and candidate proteins were inserted into the yeast prey vector pGADT7. The bait (pGBKT7) and prey (pGADT7) plasmids were co‐transformed into Y2H Gold yeast cells using the lithium acetate/polyethylene glycol/single‐stranded carrier DNA method (Coolaber) (Gietz 2014). Yeast transformants were screened ondouble dropout DDO (SD/−Trp−Leu) selective medium (Coolaber) to select yeast cells containing the desired plasmids (pGADT7 and pGBKT7). The quadruple dropout QDO (SD/−Trp/−Leu/−His/−Ade) selective medium (Coolaber) supplemented with 100 ng mL^−1^ aureobasidin A (AbA) and 20 μg mL^−1^ X‐α‐Gal was used to verify potential proteins that interact with BcGH61. The resulting colonies grown on the QDO/A/X selective medium exhibited a blue colour after being incubated at 28°C for 4 days, indicating the protein–protein interaction.

The yeast secretion assay was performed as described previously (Yin et al. 2018; Liu et al. 2024). In brief, The 5′ (200 bp) fragment (encoding SP) of *bcgh61* mature sequence was cloned into the pSUC2 vector infused with a truncated SUC2 protein and transformed into YTK12 yeast cells. Positive yeast clones were identified through screening on the CMD−W medium (0.67% yeast N base lacking amino acids, 0.075% tryptophan dropout supplement, 2% sucrose, 0.1% glucose, and 2% agar) and YPRA medium (1% yeast extract, 2% peptone, 2% raffinose, and 2% agar) plates to assess the functionality of the signal peptide after being incubated at 28°C for 3 days. To assess the secretion function of the signal peptides, a TTC‐based reduction assay was performed on yeast strains transformed with the different vectors.

### 4.14 Measurement of Electrolyte Leakage

Leaf discs (9 mm in diameter) were collected with five replicates per treatment and immersed in 5 mL of double‐distilled water at room temperature for 3 h. The initial conductivity (A) was measured using a conductivity meter (S475 SevenExcellence; Mettler Toledo). Subsequently, the samples were boiled for 30 min, cooled to room temperature, and the conductivity was measured again to obtain value B. The electrolyte leakage rate (%) was calculated as (A/B) × 100 to assess the extent of cell death in *N. benthamiana* leaves expressing different proteins. All experiments were repeated at least three times, with each measurement including a minimum of three replicates.

### 4.15 VIGS in *N. benthamiana*

VIGS was used to silence *NbBAK1* or *NbSOBIR1* as described previously (Kettles et al. 2017). The expression level of *NbBAK1* or *NbSBOIR1* in gene‐silenced *N. benthamiana* was determined by RT‐qPCR analysis (Franco‐Orozco et al. 2017). Plasmid constructs pTRV1, pTRV2:GFP (control), pTRV2:*NbBAK1*, and pTRV2:*NbSOBIR1* were introduced into *A. tumefaciens* GV3101 using electroporation for genetic transformation. *A. tumefaciens* harbouring pTRV1 and pTRV2 were suspended in infiltration buffer (10 mM MgCl_2_, 200 mM acetosyringone and 10 mM MES, pH 5.6) and then mixed at a 1:1 ratio. And the cultures infiltrated into *N. benthamiana* leaves that were maintained for 2–3 weeks in a growth chamber at 20°C. Then, *bcgh61* was expressed through *A. tumefaciens‐*mediated transient expression in the leaves of *NbBAK1‐* or *NbSOBIR1*‐silenced *N. benthamiana*. The infiltrated plants were then kept in the greenhouse at 25°C, and the cell death development was photographed 2–3 days after treatment.

### 4.16 Statistical Analysis

All experiments were repeated and yielded reproducible results. The most representative data are shown in this paper. Data are presented as means ± SE of the mean, unless stated otherwise. GraphPad Prism was used for statistical tests. ANOVA and Student's *t* test were used to analyse data significance. Asterisks or different letters in the graphs indicate statistical differences.

## Author Contributions

**Wenjun Zhu:** conceptualization, investigation, methodology, formal analysis, writing – original draft. **Ziyao Wang:** investigation, methodology, formal analysis, visualization, writing – original draft. **Can Zheng:** investigation, methodology, formal analysis. **Min Fang:** investigation, methodology, formal analysis. **Binbin Huang:** investigation, methodology, formal analysis. **Xiaofei Nie:** investigation, methodology, formal analysis, visualization. **Yong Liang:** investigation, conceptualization, visualization. **Zhaoxia Li:** investigation, methodology. **Kai Bi:** conceptualization, supervision, investigation, funding acquisition, writing – original draft, writing – review and editing, project administration, resources.

## Funding

This research was supported by the National Natural Science Foundation of China (Grant No. 32372514), Research and Innovation Initiatives of WHPU (Grant No. 2024 J02) to K.B. and Y.L. (202108280009) was funded by the China Scholarship Council.

## Conflicts of Interest

The authors declare no conflicts of interest.

## Supporting information


**Figure S1:** Protein analysis of the samples.

**Figure S2:** Bioinformatic analysis of BcGH61.

**Figure S3:** The enzymatic quadruple mutant BcGH61^H19A H181A Q190A Y192A^ (BcGH61^EM^) retains the capacity to activate plant immune responses.

**Figure S4:** NbBAK1/NbSOBIR1 signalling is not required for BcGH61‐induced cell death.

**Figure S5:** PCR validation of *Botrytis cinerea* mutant strains used in this study.

**Figure S6:** Deletion of the *bcgh61* gene does not alter colony morphology or compromise cell wall stress tolerance.

**Figure S7:** Schematic diagram of the up‐regulated genes involved in Plant‐pathogen interaction (ko04626).

**Table S1:** List of differentially expressed genes (DEGs).

**Table S2:** KEGG enrichment analysis of the DEGs upregulated in the BcGH61‐expressing line.

**Table S3:** KEGG enrichment analysis of the DEGs downregulated in the BcGH61‐expressing line.

**Table S4:** Summary of IP‐LC–MS–MS data.

**Table S5:** List of *Botrytis cinerea* strains used in this study.

**Table S6:** List of PCR primers used in this study.

## Data Availability

Sequence data of BcGH61 and NbHrBP1 are available from NCBI at https://www.ncbi.nlm.nih.gov/protein/ATZ53918.1 and https://www.ncbi.nlm.nih.gov/protein/ADM18295.1?report=genbank&log$=protalign&blast_rank=1&RID=HVXR8FNM016, with accession number ATZ53918.1 and ADM18295.1. Other data that support the findings of this current study are available from the corresponding author on reasonable request.
