# Supplementary material for: Secreted Glycoside Hydrolase BcGH61 From Botrytis cinerea Induces Cell Death by the Apoplastic Location and Triggers Intracellular Immune Perception
Source: Mol Plant Pathol. 2025 Dec 30;27(1):e70199. doi: 10.1111/mpp.70199 (PMC12754035; doi:10.1111/mpp.70199)
Supplement: Supplementary file 1 — Figure S1: Protein analysis of the samples. [file MPP-27-e70199-s006.docx]

**
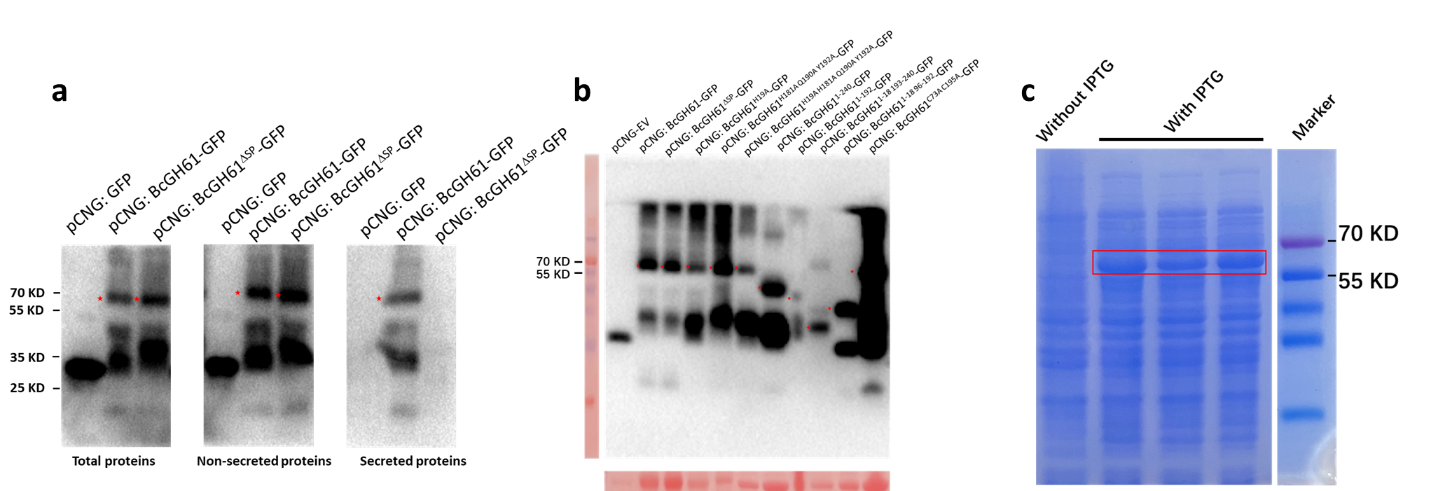
**

**Supplementary figure 1**. **Protein analysis of the samples. a, b**, Immunoblotting (IB) analysis in *N. benthamiana* leaves transiently expressing the proteins. Leaf samples were taken 2 days post infiltration with *A. tumefaciens*. **a**, Total protein extracts, apoplastic fluid fractions, and non-secreted proteins (the residual fraction following apoplastic protein extraction) were prepared and subjected to Western blot analysis. As shown in the blot, GFP-tagged BcGH61 was present in the total protein and non-secreted fractions of leaves transiently expressing either the full-length BcGH61 or BcGH61^ΔSP^. Notably, accumulation of the protein in the apoplastic fluid was observed only in leaves expressing the full-length BcGH61. α-GFP antibody was used to detect expression of the indicated constructs. Target protein products were marked with red stars. **c**, Coomassie brilliant blue (CBB) stain was used to confirm the presence of target proteins from *E. coli* lysate. Red box indicates the expected products with nonspecific bands.
