# Supplementary material for: Secreted Glycoside Hydrolase BcGH61 From Botrytis cinerea Induces Cell Death by the Apoplastic Location and Triggers Intracellular Immune Perception
Source: Mol Plant Pathol. 2025 Dec 30;27(1):e70199. doi: 10.1111/mpp.70199 (PMC12754035; doi:10.1111/mpp.70199)
Supplement: Supplementary file 2 — Figure S2: Bioinformatic analysis of BcGH61. [file MPP-27-e70199-s003.docx]

**
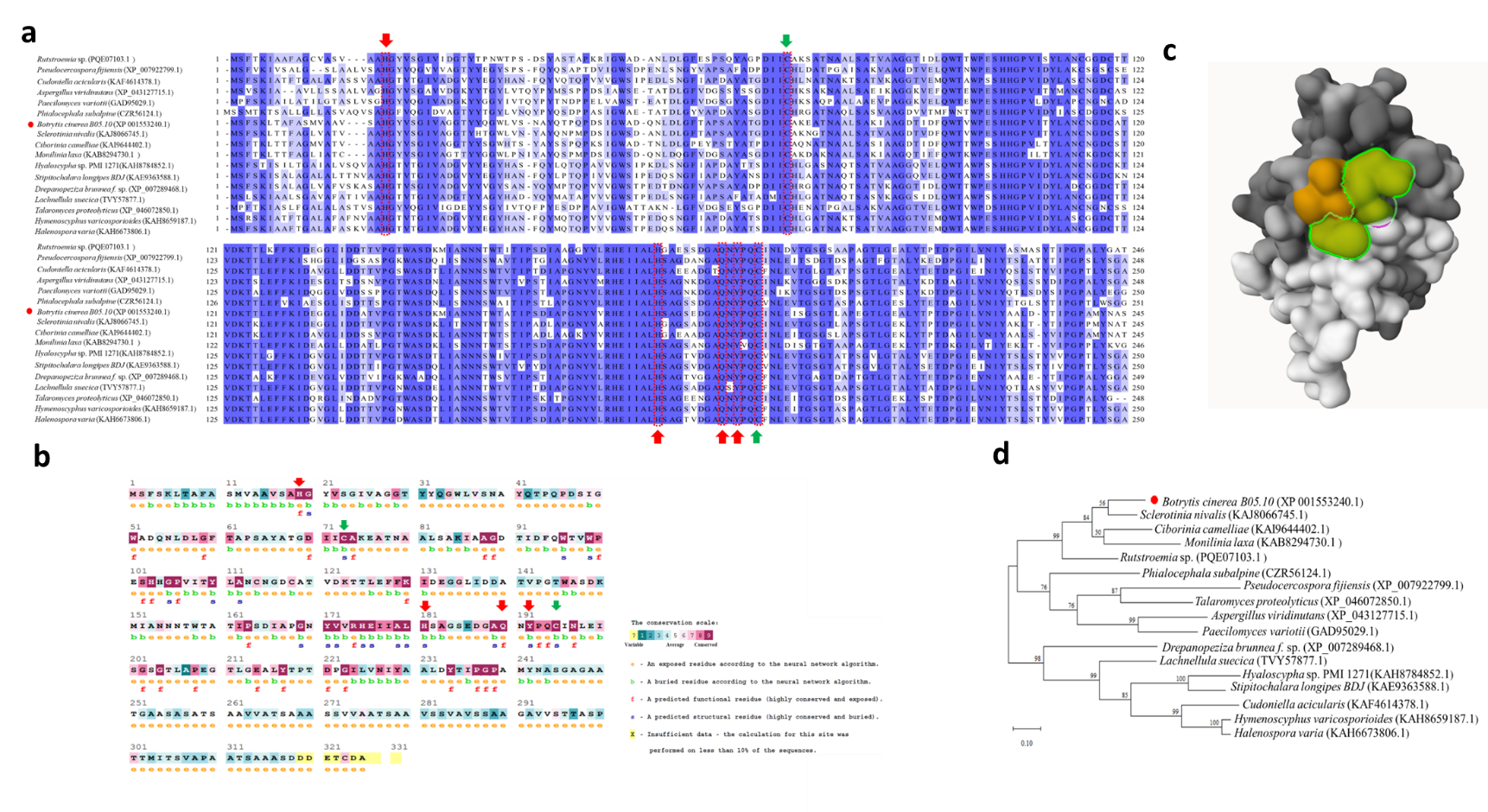
**

**Supplementary figure 2. Bioinformatic analysis of BcGH61. a,** Multiple sequence alignment (MSA) analysis of BcGH61 (highlighted in red) was performed using Clustal Omega online tool and visualized with Mview. Two conserved cysteine residues (C73 and C195) were annotated with green arrows, and catalytic pocket harboring four residues (H19, H181, Q190, Y192) were marked with red arrows. **b**, Evolutionary conservation analysis of BcGH61 was conducted using the ConSurf Server (<https://consurf.tau.ac.il/consurf_index.php>). Amino acid positions were color-coded according to conservation scores, reflecting their evolutionary importance. Structurally buried cysteine residues C73 and C195 were labeled with green arrows, and the catalytic pocket residues H19, H181, Q190, Y192, which exhibit high conservation, were marked with red arrows. **c**, The structural model generated by HHpred with integrated ConSurf-derived conservation scores was subsequently subjected to ligand-binding site prediction analysis using P2Rank (https://prankweb.cz/) to identify evolutionarily conserved catalytic pockets. The computational prediction revealed four key residues (H19, H181, Q190, and Y192) forming a putative catalytic center, which were conspicuously annotated with green circular markers in the structural visualization. **d**, A neighbour-joining tree was constructed based on the MSA result, with BcGH61 highlighted in red to indicate its evolutionary position.
