# Supplementary material for: Secreted Glycoside Hydrolase BcGH61 From Botrytis cinerea Induces Cell Death by the Apoplastic Location and Triggers Intracellular Immune Perception
Source: Mol Plant Pathol. 2025 Dec 30;27(1):e70199. doi: 10.1111/mpp.70199 (PMC12754035; doi:10.1111/mpp.70199)
Supplement: Supplementary file 3 — Figure S3: The enzymatic quadruple mutant BcGH61H19A H181A Q190A Y192A (BcGH61EM) retains the capacity to activate plant immune responses. [file MPP-27-e70199-s009.docx]

**
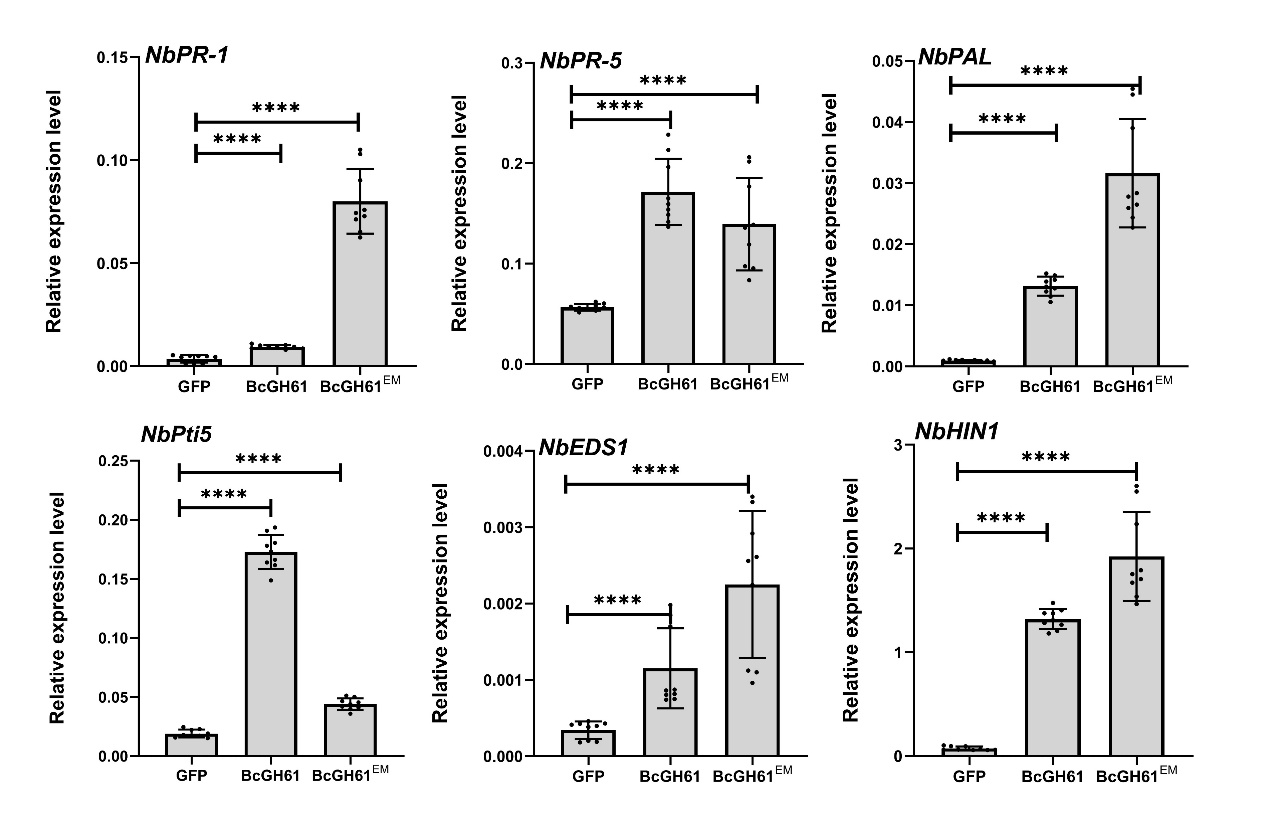
Supplementary figure 3. The enzymatic quadruple mutant BcGH61^H19AH181AQ190AY192A^ (BcGH61^EM^) retains the capacity to activate plant immune responses.** Relative expression of defense-related marker genes in *N. benthamiana* leaves transiently expressing the indicated constructs was measured. Leaf samples were harvested 2 days post-agroinfiltration. Gene expression levels were quantified by qRT-PCR and normalized to *N. benthamiana EF-1α* reference gene, with expression in GFP-expressing plants was set as control. Data represent the mean ± SD (n = 9, from three independent biological replicates, each with three technical replicates). Asterisks indicate statistically significant differences compared to the GFP control (**** *p* < 0.0001).
