# Supplementary material for: Secreted Glycoside Hydrolase BcGH61 From Botrytis cinerea Induces Cell Death by the Apoplastic Location and Triggers Intracellular Immune Perception
Source: Mol Plant Pathol. 2025 Dec 30;27(1):e70199. doi: 10.1111/mpp.70199 (PMC12754035; doi:10.1111/mpp.70199)
Supplement: Supplementary file 4 — Figure S4: NbBAK1/NbSOBIR1 signalling is not required for BcGH61‐induced cell death. [file MPP-27-e70199-s002.docx]

**
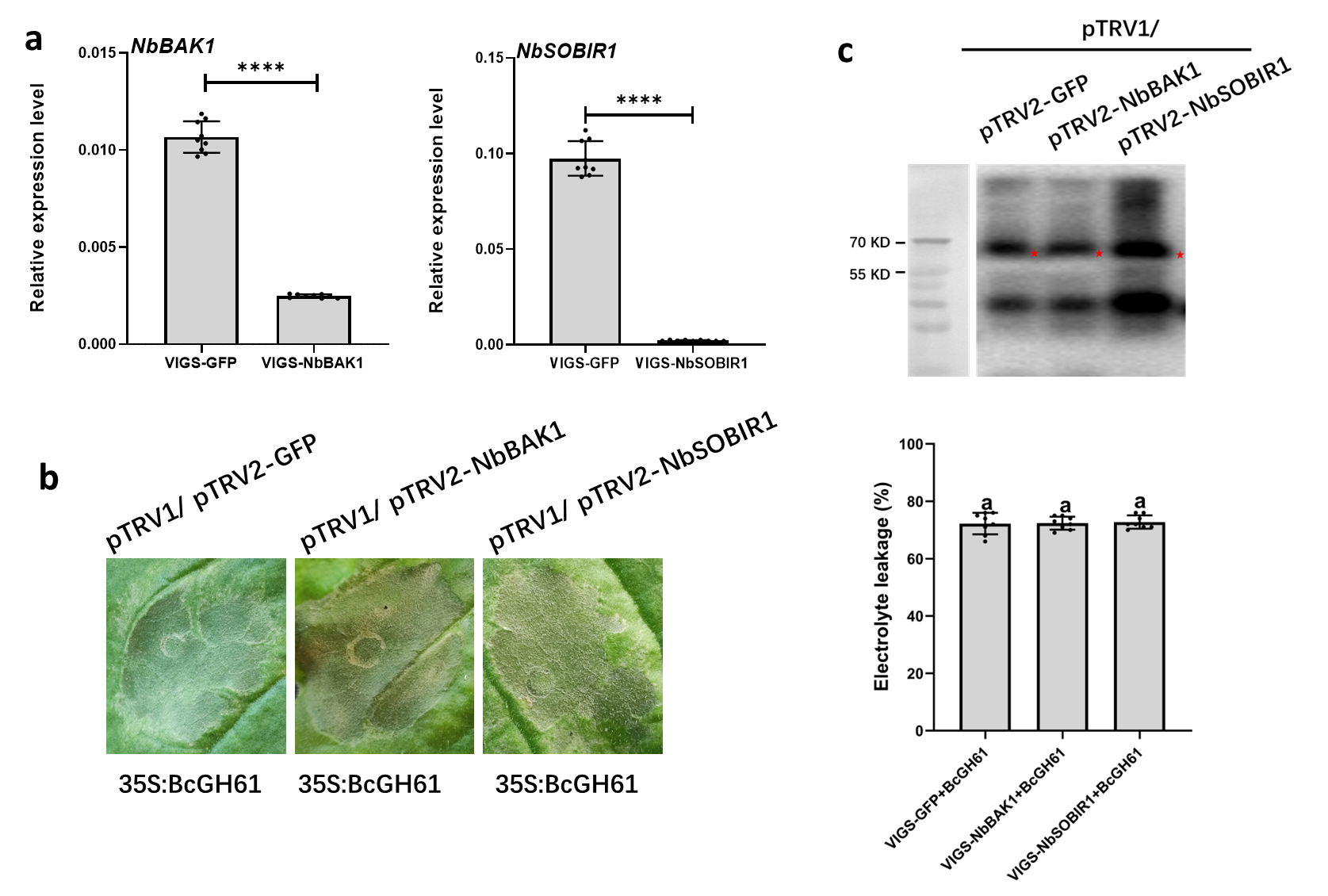
Supplementary figure 4. NbBAK1/NbSOBIR1 signaling is not required for BcGH61-induced cell death.** **a**, Silencing efficiency of *NbBAK1* and *NbSOBIR1* in TRV1-GFP, TRV2-NbBAK1, and TRV2-NbSOBIR1 plants. Relative expression is presented as the mean ± SD (n = 9) from three independent biological replicates and three technical replicates. Unpaired two-tailed Student's t test analysis. **** stand for *p* < 0.0001. **b**, Cell death phenotypes of VIGS-treated N. benthamiana leaves following transient expression of BcGH61. Photographs were captured at 3 days post-Agroinfiltration. As shown in the right panel, cell death in *N. benthamiana* leaves expressing BcGH61 was quantified by measuring electrolyte leakage. The results (mean ± SD, n = 9 from three independent experiments) are marked with different lowercase letters to indicate significant differences (*p* < 0.01, one-way ANOVA). **c**, Immunoblot analysis of BcGH61 protein accumulation in agroinfiltrated *N. benthamiana* leaves. Total proteins were extracted at 2 dpi and probed with α-GFP antibody. Target protein products were marked with red stars.
