# Supplementary material for: Secreted Glycoside Hydrolase BcGH61 From Botrytis cinerea Induces Cell Death by the Apoplastic Location and Triggers Intracellular Immune Perception
Source: Mol Plant Pathol. 2025 Dec 30;27(1):e70199. doi: 10.1111/mpp.70199 (PMC12754035; doi:10.1111/mpp.70199)
Supplement: Supplementary file 5 — Figure S5: PCR validation of Botrytis cinerea mutant strains used in this study. [file MPP-27-e70199-s013.docx]

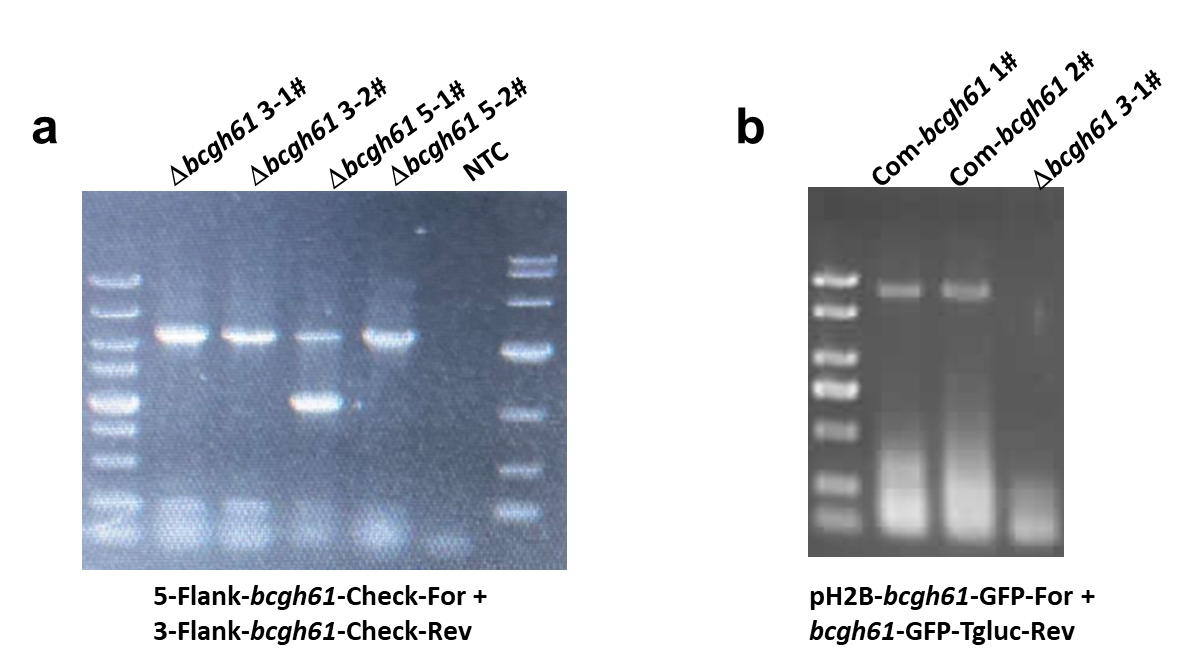
**Supplementary figure 5. PCR validation of *B. cinerea* mutant strains used in this study. a,** Genotypic analysis of *bcgh61* deletion strains Δ*bcgh61*. **b,** Confirmation of *bcgh61* complementary strains Com-*bcgh61*. Genomic DNA was extracted from each mutant strain for PCR amplification. Primers used in PCR were indicated. NTC - no template control, ddH_2_O was added as a template-free negative control.
