# Supplementary material for: Secreted Glycoside Hydrolase BcGH61 From Botrytis cinerea Induces Cell Death by the Apoplastic Location and Triggers Intracellular Immune Perception
Source: Mol Plant Pathol. 2025 Dec 30;27(1):e70199. doi: 10.1111/mpp.70199 (PMC12754035; doi:10.1111/mpp.70199)
Supplement: Supplementary file 6 — Figure S6: Deletion of the bcgh61 gene does not alter colony morphology or compromise cell wall stress tolerance. [file MPP-27-e70199-s004.docx]

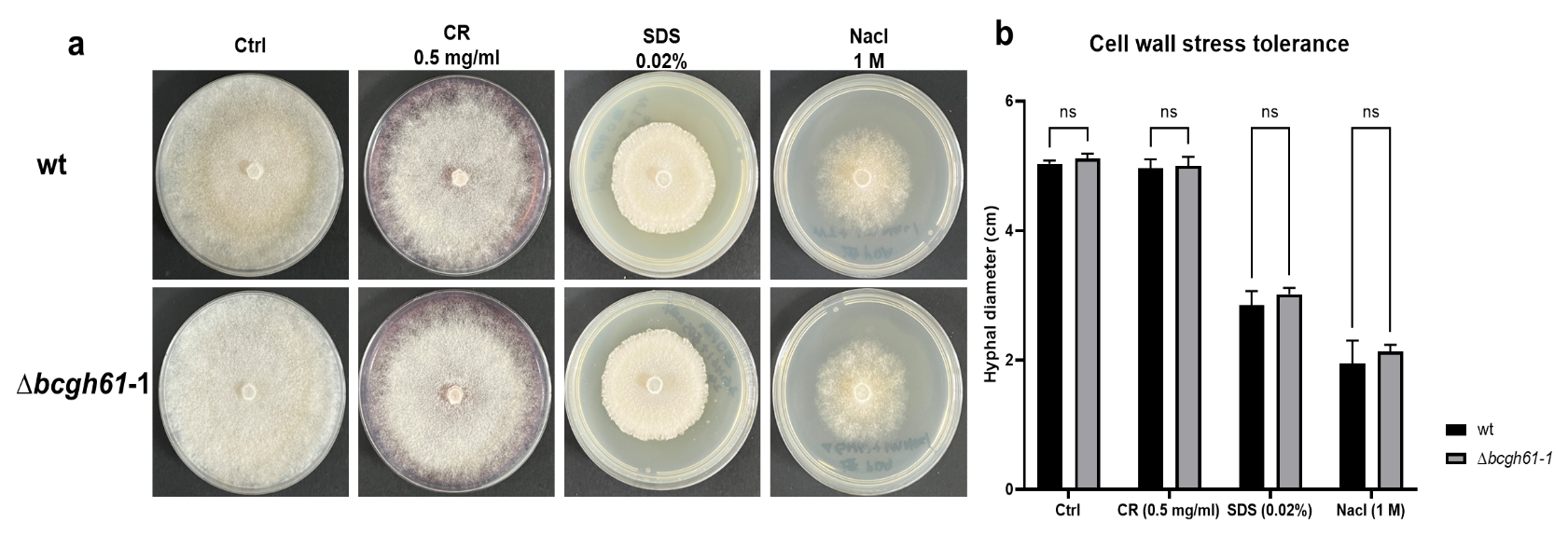
**Supplementary figure 6. Deletion of the *bcgh61* gene does not alter colony morphology or compromise cell wall stress tolerance.** Hyphal growth dynamics of the indicated strains were evaluated in PDA medium supplemented with 0.5 mg/ml Congo Red (CR), 0.02% SDS, and 1 M NaCl. **a**, Representative colony morphologies captured 2 days post-inoculation. **b**, Quantitative analysis of colony diameters. Data are presented as mean ± SD (n = 6) from three independent biological replicates. Statistical comparisons by two-way ANOVA showed no significant differences (ns: *p* > 0.05).
