# Supplementary material for: Secreted Glycoside Hydrolase BcGH61 From Botrytis cinerea Induces Cell Death by the Apoplastic Location and Triggers Intracellular Immune Perception
Source: Mol Plant Pathol. 2025 Dec 30;27(1):e70199. doi: 10.1111/mpp.70199 (PMC12754035; doi:10.1111/mpp.70199)
Supplement: Supplementary file 7 — Figure S7: Schematic diagram of the up‐regulated genes involved in Plant‐pathogen interaction (ko04626). [file MPP-27-e70199-s008.docx]

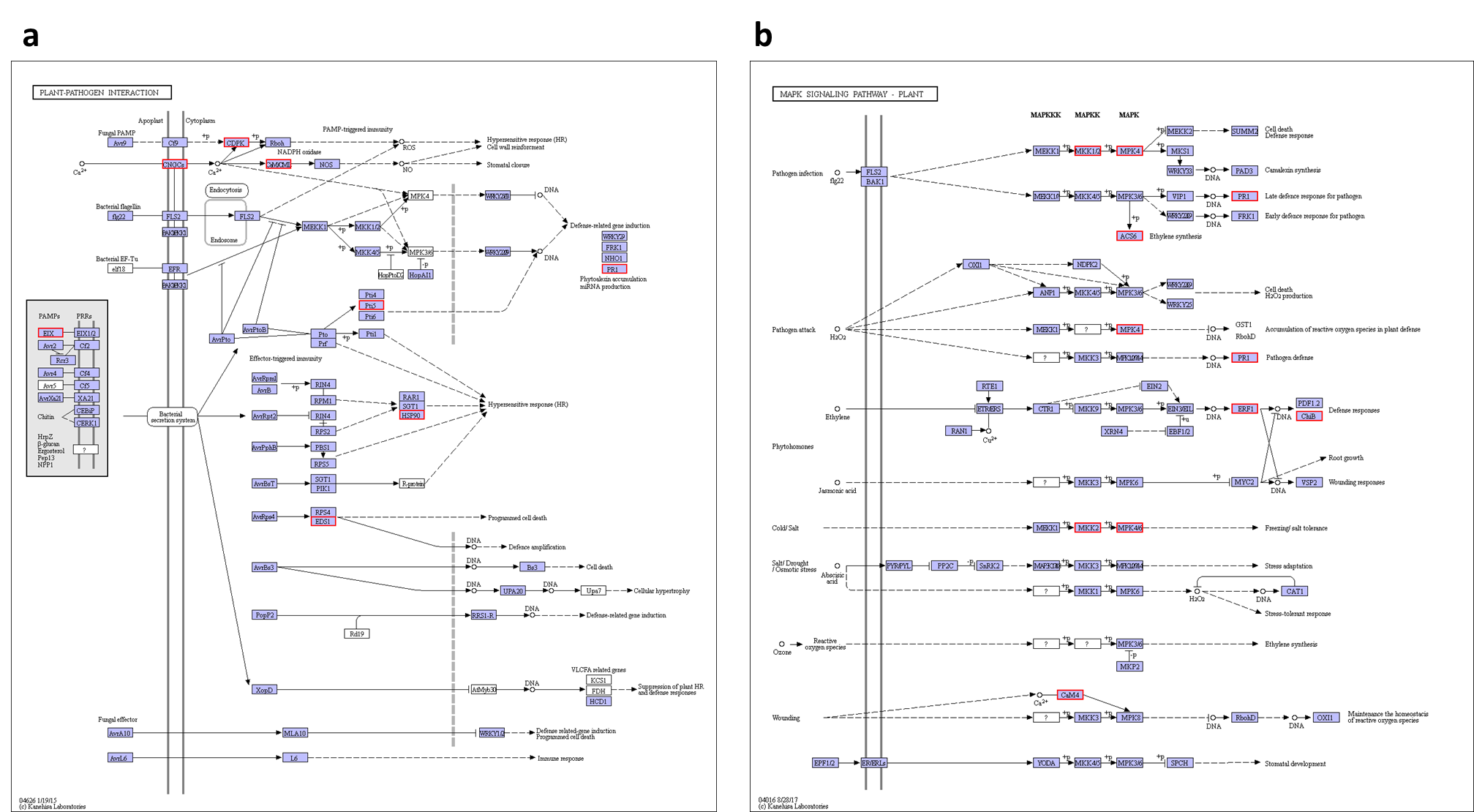


**Supplementary figure 7**. Schematic diagram of the up-regulated genes involved in Plant-pathogen interaction (ko04626) (**a**), MAPK signaling pathway – plant (ko04016) (**b**), with red knots representing the up-regulated genes.
